# Theory-Based Intervention Module on Occupational Safety and Health (TRIMOSH) in improving knowledge, attitude, and practice among food industry workers: Study protocol for a randomised controlled trial

**DOI:** 10.1371/journal.pone.0295771

**Published:** 2024-01-02

**Authors:** Rahmat Dapari, Mohd Hafizuddin Mahfot, Mohd ‘Ammar Ihsan Ahmad Zamzuri, Zaleha Md Isa, Mohd Rohaizat Hassan, Nazri Che Dom, Syed Sharizman Syed Abdul Rahim

**Affiliations:** 1 Department of Community Health, Universiti Putra Malaysia, Serdang, Malaysia; 2 State Department of Health Negeri Sembilan, Seremban, Negeri Sembilan, Malaysia; 3 Department of Community Health, Universiti Kebangsaan Malaysia, Cheras, Malaysia; 4 Faculty of Health Sciences, Universiti Teknologi MARA, Bandar Puncak Alam, Malaysia; 5 Public Health Medicine Department, Faculty of Medicine and Health Sciences, Universiti Malaysia Sabah, Kota Kinabalu, Malaysia; Pharmaceutical Services Division, MALAYSIA

## Abstract

**Introduction:**

The significant contribution of the food and beverage industry to Malaysia’s Gross Domestic Product is projected to increase in the upcoming years. With the industry’s expansion, the demand for workers on food premises would also continuously increase. The food industry workers are exposed to risks arising from physical, chemical, biological, ergonomic, and psychosocial hazards while performing their duties. Thus, it is essential for these workers to be equipped with proper knowledge, attitude, and practices (KAP) in safety and health.

**Aims:**

This study aims to develop and evaluate the effectiveness of the safety and health programme TRIMOSH (**T**heo**r**y-Based **I**ntervention **M**odule on **O**ccupational **S**afety and **H**ealth) in improving the knowledge, attitude, and practice among food industry workers.

**Methods:**

TRIMOSH intervention study is a two-arm randomised, single-blinded, controlled, parallel trial that will be conducted among food industry workers in Selangor, Malaysia. In a partnership with Food Handler Training Schools in Selangor, 10 pairs of Food Handler Training Schools with 12 participants per group (n = 240) will be recruited for balanced randomisation intervention and control conditions. Furthermore, data collection of all participants was conducted at four time points: baseline (T_0_), immediately (T_1_), one month (T_2_), and three months (T_3_) post-intervention. Generalised Linear Mixed Model (GLMM) will be conducted to determine the effects of intervention within and between study groups. Subsequently, the primary outcomes increase the knowledge, attitude, and practice (KAP) of safety and health at food premises. Clinical Trial Registry registration was approved by the ClinicalTrials.gov committee on October 2022 with the ClinicalTrials.gov Identifier: NCT05571995. This study has also been approved by the Ethics Committee for Research Involving Human Subjects of Universiti Putra Malaysia (JKEUPM-2022-346). All participants are required to provide consent prior to participation.

**Conclusions:**

The characteristics of the respondents are expected to show no difference between the groups. It is hypothesised that TRIMOSH is effective in improving the knowledge, attitude, and practices of food industry workers in Selangor. The results will be reported and presented in international peer-reviewed journals, conferences, and other platforms. In addition, the TRIMOSH programme will be offered at the national level by the relevant authorities for the benefit of food industry workers.

## Introduction

Food industry workers are defined as individuals who are directly involved in food preparation, come into contact with food or food contact surfaces, and handle packaged or unpackaged food, or appliances, on any food premises [1]. Since 1996, the Ministry of Health Malaysia (MOH) has been organising food handler training courses to provide exposure and awareness to food industry workers regarding food safety, hygiene, self-hygiene, and premises hygiene, mainly to prevent food poisoning incidents in the country [2]. Subsequently, under the Food Hygiene Regulations 2009, all food industry workers are mandated to attend Food Handler Training Courses organised by any schools or institutes that have been certified by the Ministry of Health (MOH) Malaysia, namely Food Handler Training Schools. Under this regulation, any food industry workers who fail to undergo training or obtain a Certificate of Food Handlers Training shall be liable to a fine or compound not exceeding RM10,000 or imprisonment for a term not exceeding two years [3].

Similar to any other occupations, the occupational safety and health of food industry workers in Malaysia are generally governed and regulated by the Occupational Safety and Health (OSHA) Act 1994 (Act 514) [4]. The spirit of the act is to secure the safety, health, and welfare of individuals at work, protecting them from any risk or health implications associated with their activities at work and preventing occupational diseases. In this case, occupational disease is defined as any disease contracted due to exposure to risk factors arising from activities at work [5]. However, no occupational safety and health training module has been required or made compulsory by the government to equip the food industry workers with appropriate knowledge, attitude, and practice of occupational safety and health and to prevent injuries and illnesses related to their work.

The food and beverage industry has contributed a significant amount to Malaysia’s Gross Domestic Product [6]. This contribution is projected to increase in the upcoming years. With the expansion of the industry, the demand for workers on food premises will also continuously increase. The workforce mainly comprises workers from the food processing sector, food distribution sector, and food service sector on the food premises. As these workers fulfil their duties, they are exposed to risks arising from physical, chemical, biological, ergonomic, and psychosocial hazards. Additionally, the number of workers has increased, which may lead to an increase in the number of accidental injuries or occupational diseases related to the food and beverage industry. Thus, it is crucial for them to be equipped with proper knowledge, attitude, and practice (KAP) towards safety and health.

At the global level, 14.1% of nonfatal occupational injuries and illnesses involving days away from work (DAFW) in the private industry in 2020 are attributed to the manufacturing industry, which includes the food and beverage sector. The other 7.2% of the injuries were attributed to food services [7]. Notably, the highest number of nonfatal occupational injuries and illnesses involving DAFW in the private industry impact the food industry workers within the industries. This condition led to 22% of total reported cases or approximately 0.3 million cases [8]. It was reported that the highest contributors of injuries and illnesses for known classified cases among food industry workers are cuts, lacerations and punctures (15%). This was followed by sprain, strains, and tears (15%), soreness and pain (13%), thermal burn (8%), bruises and contusions (5%), fractures (4%), multiple traumatic injuries (1%), chemical burns and corrosions (1%), carpal tunnel syndrome (1%), amputation (0.4%), and tendonitis (0.06%). However, several reported cases were non-classifiable, which contributed to approximately 35% of the total reported cases among food industry workers [9].

Notably, the highest number of injuries and illnesses experienced by food industry workers was caused by exposure to harmful substances or environments (34%), followed by contact with objects or equipment (22%), falls, slips, and trips (21%), overexertion and bodily reaction (19%), transportation incidents (2%), violence and other injuries by persons or animal (1%), other events or exposures (0.4%), and fires and explosions (0.09%) [10]. The top ten highest reported incidences of occupational injuries and illnesses among food industry workers were observed among those who worked in food preparation and serving-related occupations (25%), food preparation workers (10%), food and beverage serving workers (9%), food processing workers (6%), cooks (5%), fast food and counter workers (5%), food preparation workers (5%), supervisors of food preparation and serving workers (4%), butchers and other meat, poultry and fish processing workers (3%), and other food preparation and serving related workers (3%) [9].

Based on local statistics in Malaysia, the manufacturing industry, which includes the food and beverage sector, is the most significant contributor to all confirmed occupational poisoning and disease cases at 82.3%. On the other hand, the percentages of confirmed cases from hotels and restaurants, including wholesale and retail trades amounted to 0.5% and 0.3% of total confirmed cases, respectively. In terms of accidental injury at the workplace categorised by sector, the Department of Occupational Safety and Health (DOSH) Malaysia reported an increasing trend of accidents among food industry workers annually. In 2020 only, 62% of accidents with temporary disability, 84% of accidents with a permanent disability, and 34% of deaths reported to DOSH took place in the manufacturing industry. Apart from that, 2% of accidents with temporary disability, 0.4% of accidents with permanent disability, and 0.7% of deaths reported by DOSH occurred in the hotel and restaurant industry. Following that, wholesale and retail trades reported 2% of accidents with temporary disability, 0.4% of accidents with permanent disability, and 0.4% of deaths [5].

The Ministry of Health has introduced the compulsory Food Handler Training Course for food industry workers. This course is formalised and standardised mainly on the aspects of food introduction, food hygiene, food safety, and critical factors of food poisoning. However, the aspect of workers’ safety and health is not covered, given that the safety and health of food industry workers fall under DOSH authority. Based on the available data on occupational poisoning, occupational diseases, and accidents that lead to temporary and permanent disability among food industry workers, training programmes focusing on workers’ safety and health, such as the Theory-based Intervention Module on Occupational Safety and Health (TRIMOSH), are appropriate to be conducted for food industry workers. TRIMOSH is expected to improve the knowledge, attitude, and practice of safety and health among food industry workers at food premises and prevent safety and health issues.

### Study aims

The overarching objective is to develop and evaluate the effectiveness of safety and health programmes for Food Industry Workers to enhance their knowledge, attitude, and practice. The intervention is made through the **TRIMOSH** (**T**heo**r**y-Based **I**ntervention **M**odule on **O**ccupational **S**afety and **H**ealth) programme. It is hypothesised that the intervention group would demonstrate beneficial effects related to the intended outcomes of safety and health (knowledge, attitude, and practice score).

## Methods

### Clinical trial registry and ethics approval

Clinical Trial Registry registration was approved by the ClinicalTrials.gov committee on October 2022 with the ClinicalTrials.gov Identifier: NCT05571995. This study has been approved by the Ethics Committee for Research Involving Human Subjects of Universiti Putra Malaysia (JKEUPM-2022-346).

### Overview and study duration

**Fig 1:**
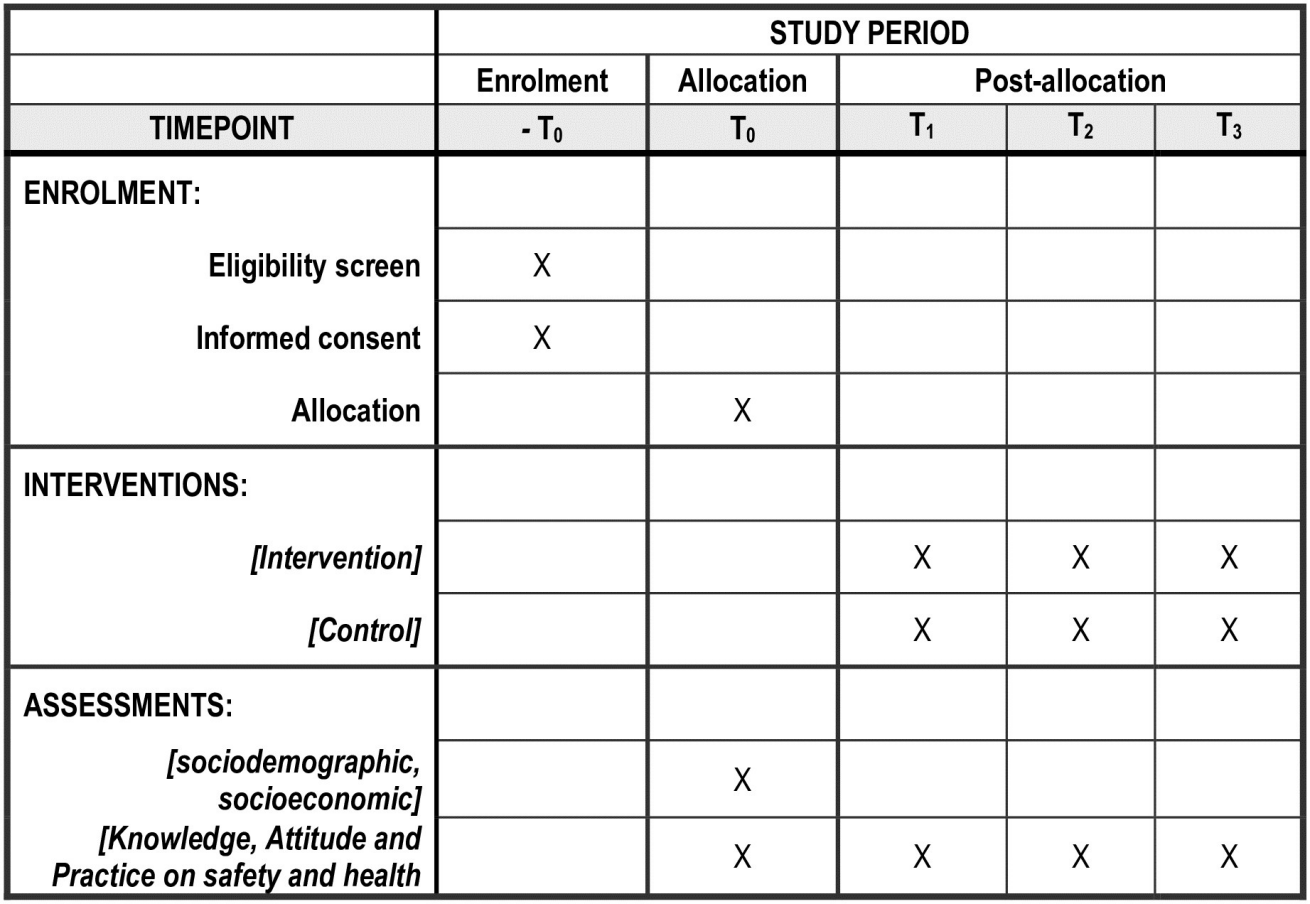
Schedule of enrolment, intervention, measurement, and assessment.

**Fig 2:**
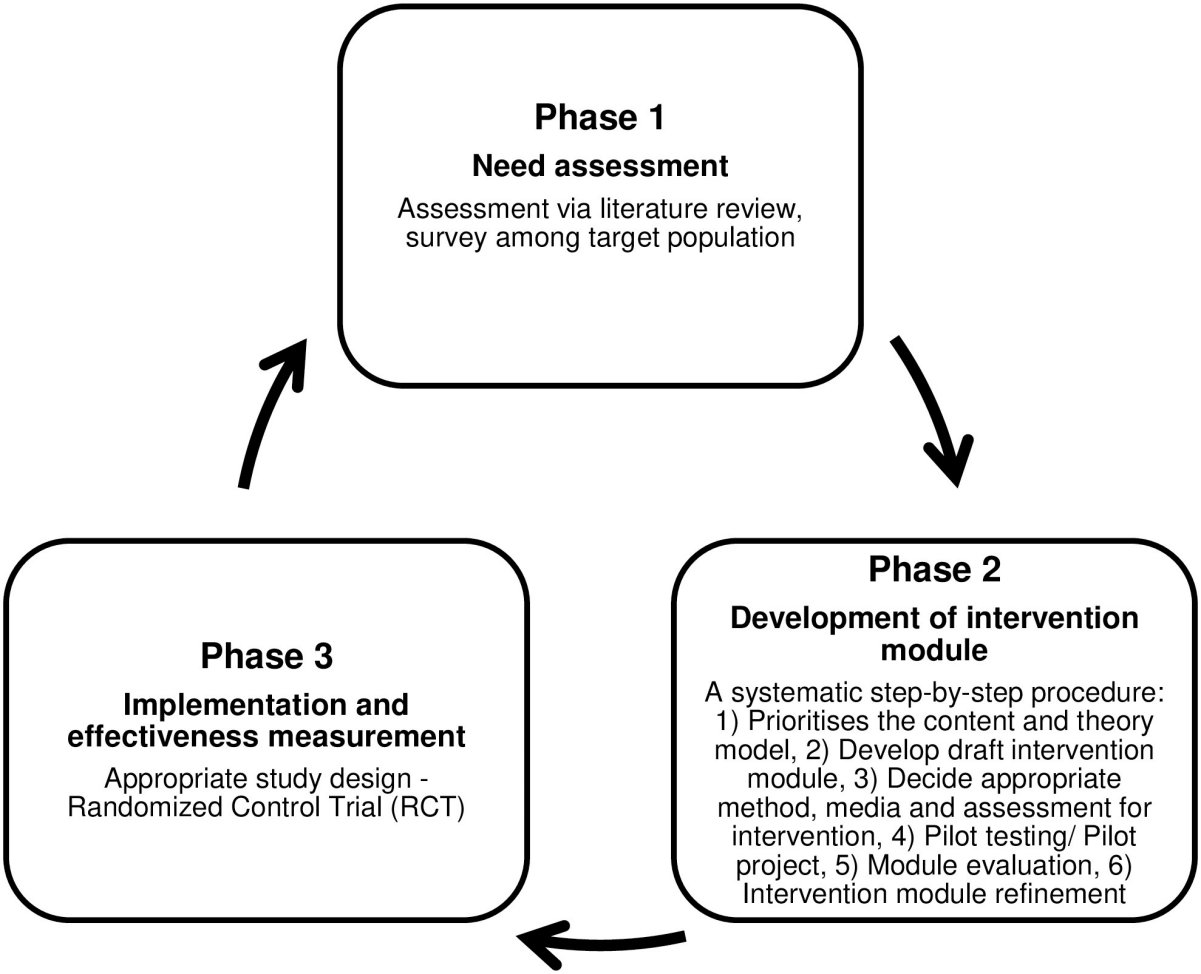
Three Phases of Intervention Module Development (3P-IMD).

This research protocol involves the process of development, implementation and effectiveness assessment, and dissemination of the Theory-based Intervention Module on Occupational Safety and Health (TRIMOSH) among food industry workers. It will be conducted for 24 months, from October 2022 to September 2024. Fig 1 presents the schedule of enrolment, intervention, and measurement to be used in this study, including the corresponding time of assessment. The study consists of three phases of intervention module development (3P-IMD) as shown in Fig 2, namely **Phase I** (need assessment), **Phase II** (intervention module development), and **Phase III** (implementation and effectiveness assessment of intervention module). Data collection for randomized controlled trial (RCT) will be performed on 1^st^ December 2023.

### Phase I: Need assessment

In this cross-sectional study, the need assessment of the intervention was conducted through a literature review and survey among food industry workers in Selangor. This survey involved an investigation into the prevalence of occupational injury and possible associated factors. Assessment of current knowledge, attitude, and practice on safety and health was also collected and measured. The demand for occupational training among food industry workers was also measured [11].

### Phase II: Intervention module development

A systematic step-by-step procedure will be conducted to design and develop a Theory-based Intervention Module on Occupational Safety and Health (TRIMOSH), as shown in Table 1. The intervention module development consists of six steps, as shown in the Table 1. To ensure that the objectives of TRIMOSH and the Food Handlers Training Course do not overlap, the participants and subject matter experts during the TRIMOSH development will be reminded that TRIMOSH focuses on Food Industry Workers’ safety and health, while the Food Handler Training Course focuses on food safety and hygiene.

**Table 1:** A systematic step-by-step procedure for intervention module development.

Step	Objective	Activities
1	Prioritises safety and health problems	Subject matter expert (SME) consensus from various agencies such as the Ministry of Health, NIOSH, DOSH, SOCSO, Ministry of Higher Education, and Food Industry Workers consensus Fuzzy Delphi method will be used to rank and prioritise the safety and health problems
2	Develop prototype intervention module focusing on solution and target group	Subject Matter Expert (SME) consensus and Food Industry Workers (FIW) consensus Fuzzy Delphi method will be used to rank and prioritise the safety and health solution based on selected priorities of safety and health problems
3	Decide appropriate method and media for intervention and develop an assessment	Subject Matter Expert (SME) consensus and Food Industry Workers (FIW) consensus Fuzzy Delphi method will be used to rank and prioritise the appropriate method and media for intervention
4	Pilot testing	The target trainers for TRIMOSH include Medical Doctors (MD), Occupational Health Doctors (OHD) and Safety and Health Officers (SHO). The target trainees are Food Industry Workers in Selangor, Malaysia. A total of 30 food industry workers will be involved in the pilot test
5	Module evaluation	Context, Input, Process, and Product (CIPP) evaluation model will be used to evaluate the intervention module
6	Intervention module refinement	Incorporated feedback from the pilot training and module evaluation to finalise intervention modules

**Fig 3:**
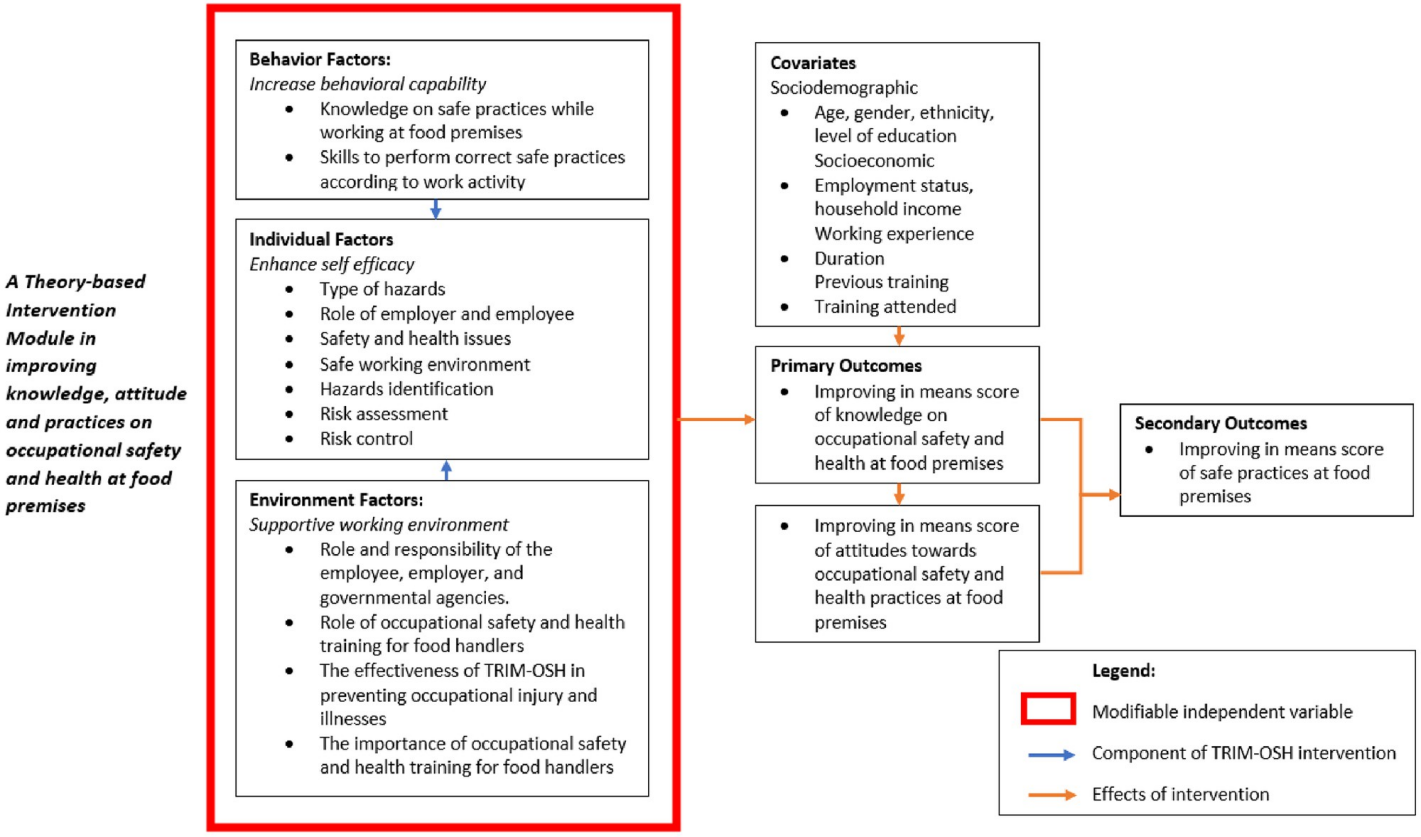
Conceptual framework of TRIM-OSH based on Social Cognitive Theory (SCT) to improve knowledge, attitude, and practice on occupational safety and health among food industry workers in Selangor.

The acceptance of the food industry workers and stakeholders to TRIMOSH and the duration and method of delivery will be identical to the Food Handler Training Course, which is a one-day face-to-face training course. The Social Cognitive Theory (SCT) has been deemed appropriate in the development of a TRIMOSH module in this study as it offers a strong construct on personal factors, environmental factors, and behavioural factors on occupational safety and health among food industry workers, as shown in Fig 3. Established in the 1960s by Albert Bandura, a well-known psychology professor, SCT provides an understanding of how human beings are dynamically influenced by their surroundings. It also discusses how human behaviour is regulated through observational learning and modelling processes, including the impact of self-efficacy on behaviour development [12].

### Phase III: Implementation and effectiveness assessment

The TRIMOSH trial is a two-arm cluster randomised, single-blinded, controlled, and parallel trial. Upon the partnership with Food Handler Training Schools in Selangor, 10 pairs of Food Handler Training Schools will be recruited for balanced randomisation of groups to intervention and control conditions. One cluster is defined as one comparable Food Handler Training School that fulfils the inclusion and exclusion criteria.

### Sample size

The sample size calculation in this study was based on the targeted primary outcomes, such as knowledge, attitude, and practice of safety and health. A previous power analysis specified an enrolment target of 20 clusters of six participants per condition. Provided that the average attrition rate in previous intervention studies was 20%, conservative estimation will be conducted on the attrition at 20% for each testing time point when the target sample size is designed. Given that the total time points in this study requirement are three times follow up after the baseline data collection, the required sample size comprises 20 clusters of 12 participants per group (N = 240). To encourage retention, lottery-style drawings will be carried out for participants who complete each data collection phase. Retention bonus pay will be offered for those who complete all four data collection phases.

### Sampling method and randomisation

Cluster randomisation will be conducted in this study, followed by intervention based on cluster sampling to avoid contamination between intervention and control groups. Specifically, cluster sampling will be carried out to select the food handler training schools, followed by randomisation of clusters to intervention and control groups. Subsequently, simple random sampling will be performed to select participants from each chosen cluster.

### Randomisation of the clusters

**Fig 4:**
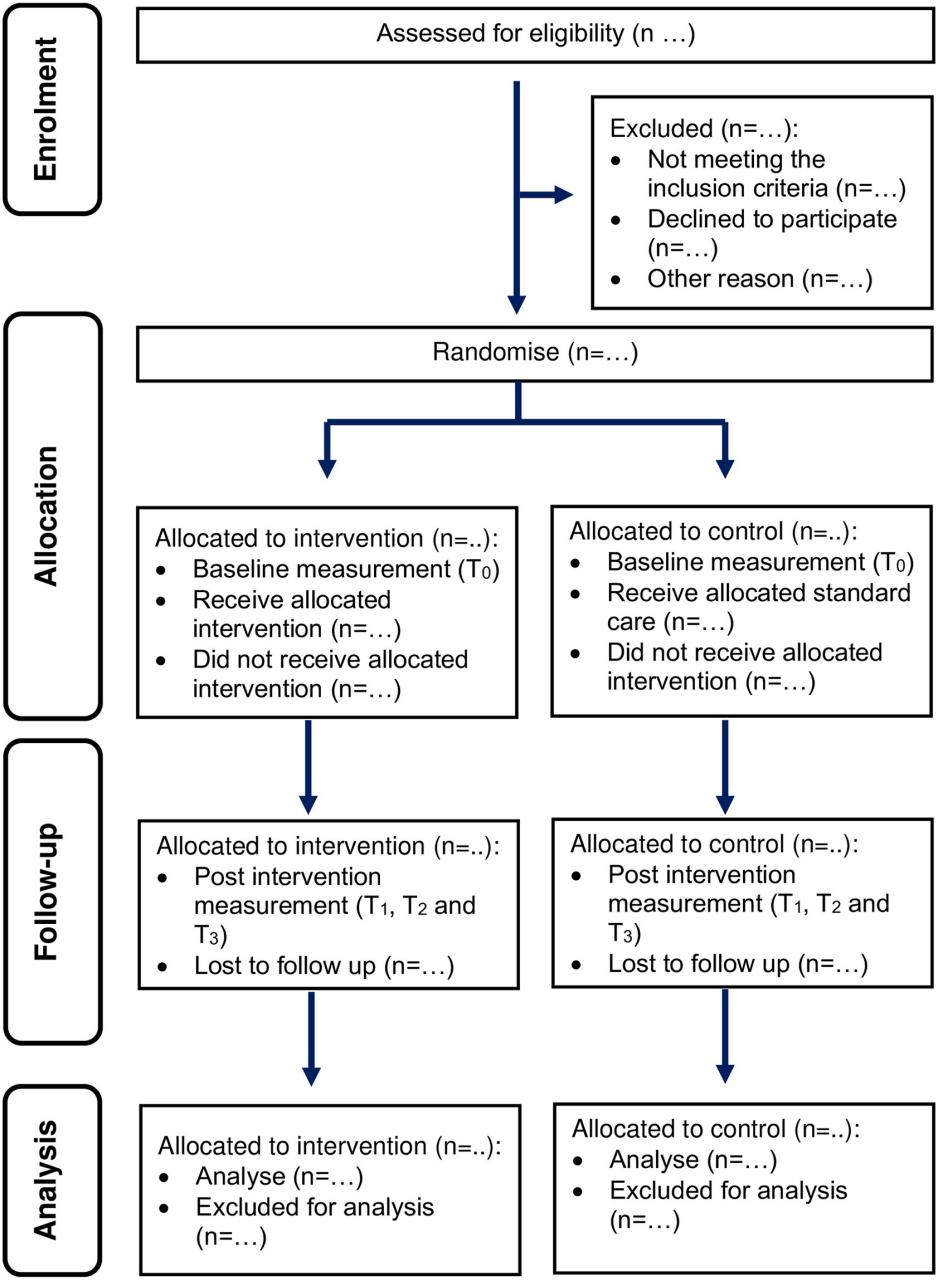
Flow diagram of study conduct based on CONSORT statement.

The list of Food Handler Training Schools will be screened for eligibility criteria. A random allocation sequence will be generated to allocate 20 Food Handler Training Schools to intervention or control groups. The randomisation sequence will be generated using software from the web page titled “Create a block randomisation list” (Sealed Envelope Ltd, 2019). The sequence generation and allocation concealment process will be conducted by the main investigator. On the other hand, the implementation of the study involving the enrolment of Food Handler Training Schools, opening the envelopes, and administration of intervention will be conducted by a trained field researcher on-site based on the suggestions by the CONSORT statement [13], as shown in Fig 4. Moreover, the permuted block randomisation will be used to allocate Food Handler Training Schools into either the intervention or control group. This method is employed to ensure that a closely balanced number of schools will be allocated to each arm of the study. In addition, variable block sizes will be adopted to avoid the prediction of the allocation process of Food Handler Training Schools.

### Simple randomisation for participants

To fulfil the eligibility criteria, the list of Food Handler Training Course trainees will be screened based on the registration number from each selected Food Handler Training School. The individuals who do not fulfil the eligibility criteria will be removed from the list. Subsequently, the cleaned trainee registration list will be arranged in ascending order, followed by the distribution of numbers to the trainees starting from one to the total number of the sample population. The next step is a simple random sampling conducted using an Excel random number generator to recruit participants based on the sample size required for each cluster.

### Blinding

This study will employ the single blinding method, in which the participants will not be aware of their group status. This type of blinding aims to minimise performance bias, which is also known as the Hawthorne effect where the participants may change their responses or behaviour if they are aware of the group that they will be allocated to. However, it is not possible to blind the field researchers as they are responsible for delivering the intervention module to the selected participants.

### Inclusion and exclusion criteria

The inclusion criteria for Food Handler Training Schools include the schools approved by the Ministry of Health, while the exclusion criteria include those that have not been actively conducting the “Food Handler Training Course” for the past 12 months. The inclusion criteria for the participants are the food industry workers aged 18–65 years old and registered under Food Handler Training Schools. Meanwhile, the exclusion criteria comprise those who are not able to read and write in English and Malay, not working in any food premises during the study period, and are non-Malaysian residents.

### Standard intervention

The intervention group will go through TRIMOSH after completing the Food Handler Training Course while the control group will receive the Food Handler Training Course as per the usual current practice. The TRIMOSH intervention module will be carried out face-to-face by one trained investigator in a class-setting environment. To ensure that the standard intervention is performed on the participants, the main investigator will carry out pre-recorded video, briefing, and serial supervision throughout the intervention period.

### Data collection

All baseline data will be collected prior to the Food Handler Training Course. As for the intervention group, T_0_ will be collected before the Food Handler Training Course, and T_1_ will be collected after the Food Handler Training Course and TRIMOSH intervention. In the case of the control group, T_0_ will be collected before the Food Handler Training Course, while T_1_ will be collected after the Food Handler Training Course. Overall, T_2_ and T_3_ for intervention and control groups will be collected within one month and three months, respectively.

### Data analysis

Data will be compiled and analysed using Statistical Package for the Social Sciences (IBM SPSS, Version 26.0). Prior to the analysis, the normality of data will be checked graphically and statistically. Descriptive statistics using continuous data will be presented using mean (Standard Deviation, SD) if normally distributed and median (interquartile range, IQR) data are not normally distributed. Furthermore, categorical data will be presented in frequency and percentage. If the criteria for normally distributed data are fulfilled, parametric tests will be used for further analysis. In contrast, non-parametric tests will be used if the data are not normally distributed. Missing values and irrelevant answers will also be managed.

Generalised Linear Mixed Model (GLMM) will be conducted to determine the effects of sociodemographic factors (age, gender, ethnicity, level of education), socioeconomic factors (employment status, household income), and working experience (duration) on knowledge, attitude, and practice (KAP) of occupational safety and health among food industry workers at food premises pre- and post-intervention within and between study groups. Further analysis in this study will determine the effects of the SCT construct components as the base of the theory-based intervention module, namely behaviour, individual, and environmental factors on the KAP of occupational safety and health among these food industry workers.

Fixed and random effects will be determined in this study. Specifically, the fixed effects are the factors where the only level under consideration is continued in coding the effects. For example, both male and female genders fall under the factor of sex, while other examples including different ethnicities fall under the factor of ethnic group. Meanwhile, random effects are the factors with random effects, which are assumed to be drawn randomly based on a distribution during the data-generating process. The study sample is a random sample of the target population of food industry workers at food premises. The KAP score of the Food Industry Workers is required to be measured a few times.

GLMM can incorporate both fixed and random effects. Differences between individuals’ responses at baseline (intercept) across the subjects are allowed by releasing the random effect of intercept. Similarly, the release of the random effect of a linear slope allows the changes in the responses to differ across subjects over time. The incorporation of the random effects in the model allows GLMM to provide estimated parameters (fixed effect) and individual variability of these parameters around the population trend [14].

In result interpretation, all results of log-transformed data are considered underlying data transformation. The confidence interval will be set at 95% for mean estimations. Furthermore, the level of significance, alpha (α), will be set at 0.05. A p-value of lower than 0.05 pertains to the decision rule. The dependent variables measured in this study are the outcomes (primary and secondary) and effectiveness of the SCT constructs as the base of theory-based intervention modules in occupational safety and health training among food industry workers.

In addition to GLMM, multivariable analysis using Generalised Estimating Equations (GEE) will be explored to determine the effect of the intervention on the primary and secondary outcomes after adjusting for the covariates. Notably, the GEE is a powerful statistical strategy that appropriately compensates for cluster effects by employing an interchangeable correlation structure that recognises and adjusts for within-cluster correlation. The use of GEE enables the analysis to generate reasonable estimates of the intervention’s influence on the outcome variable. At the same time, the correlation between the data within the same clusters is considered by fitting this GEE model, which verifies the validity of the cluster trial analysis in this study. The effectiveness of the intervention will be based on the trial group and timepoint interaction result. Following that, the effect of the intervention on the changes in outcome measures will be determined after the intervention period. The GEE will also be applied during our analysis to account for the intrinsic cluster structure in the dataset.

#### Missing data

Assuming that the data are missing at random, multiple imputation techniques will be used to replace missing data. It will be assumed that the data is missing at random if no participant demographics or primary outcomes are correlated. Sensitivity analysis over a range of missing data mechanisms will be performed as required. In this study, only sporadic missing data are expected to occur as the research staff will be present to supervise data collection and identify potential problems with missed questions and others. Missing values will go through multiple imputed using available covariates by sequential imputation. This approach allows an optimal use of available data in analysis involving change measures.

#### Early withdrawal/dropouts

Loss to follow-up in both the intervention and comparison groups is expected to be low. A conservative estimate of this attrition rate amounts to 20% in the study period. Intent-to-treat analyses will be applied during the analysis. The follow-up data will be collected at each time point from individuals, followed by an analysis of the baseline demographics to determine the occurrence of differential dropout.

### Ethics approval and consent to participate

This study protocol and all its procedures have been approved by the Ethics Committee for Research Involving Human Subjects of Universiti Putra Malaysia (JKEUPM-2022-346). Clinical Trial Registry registration was approved by the ClinicalTrials.gov committee on October 2022 with the ClinicalTrials.gov Identifier: NCT05571995. All participants will be informed that their enrolment will not affect their relationship with their employer in any way. Consent from each participant will be obtained using a consent form upon the agreement to participate in the study before answering the baseline questionnaire. Participants are also allowed to withdraw at any point during the study.

### Study instrument

Two study instruments will be used in this study: (i) the TRIMOSH intervention module and (ii) the questionnaire. A self-administered questionnaire will be distributed to respondents in English and Malay. The questionnaire section related to knowledge, attitude, and practice will be developed in phase II, which is in line with Subject Matter Expert (SME) and food industry workers. At this stage, the Fuzzy Delphi method will be used to rank and prioritise the appropriate assessment items to measure the effectiveness of TRIMOSH.

#### TRIMOSH intervention module

The TRIMOSH intervention module’s step-by-step development procedure has been illustrated in Phase II.

#### Questionnaire

Content validity of the questionnaire will be assessed by Subject Matter Experts (SME) that comprise a public health medicine specialist, family medicine physicians, health educators, occupational health doctor, and occupational-related officer. This evaluation will be performed by various agencies, the Ministry of Health (MOH), National Institute of Occupational Safety and Health (NIOSH), Department of Occupational Safety and Health (DOSH), Social Security Organisation (SOCSO), and Ministry of Higher Education (MOHE). The Content Validation Ratio (CVR) will be calculated to assess the acceptability of the questionnaire. Furthermore, the comments and feedback by the Subject Matter Experts (SME) will be taken into consideration to make further corrections to the questionnaire. In addition, the Content Validity Index (CVI) will be calculated by the assigned experts to assess the relevance and clarity of the questionnaire. This will be followed by the calculation of the CVI (I-CVI) and the scale average CVI (S-CVI/Ave). Subsequently, the questionnaire will be modified based on the agreement level of the experts on the calculated CVI.

The reliability of the instrument will be measured using the internal consistency method calculated in IBM SPSS (Version 26). The sample size for the reliability study of the instrument will be calculated using a web-based sample size calculator available through the link https://wnarifin.github.io/ssc_web.html. Following that, respondents will be recruited to achieve the sample size. All respondents recruited for the reliability test of the instrument will be excluded from the main study. Depending on the type of scale, Cronbach’s alpha coefficient or Kuder-Richardson coefficient for each scale will be calculated to determine the measure of internal consistency of the scale. As for the items measured in the Likert scale, Cronbach’s alpha will be used to measure the internal consistency. On the other hand, the internal consistency for the dichotomous response will be measured using Kuder-Richardson’s 20 formula to determine the internal consistency of the instrument.

### Dissemination

Research dissemination, knowledge translation, and knowledge transfer will be conducted through three methods. The first method builds on networking among stakeholders at the national level to shape and broaden the health policy and improve the safety and health of food industry workers for wider dissemination on the national level. The second method is the engagement with Medical Doctors (MD), Occupational Health Doctors (OHD), and Safety and Health Officers (SHO) to inform them about the progress and emerging research findings. This is followed by conducting the trainer (TTT) programmes to expand the TRIMOSH implementation nationwide. The third method is the dissemination of the findings through traditional academic routes of conferences and peer-reviewed publications.

### Consent for publication

Consent from the participants for publication will be obtained. In this case, no personnel or company information will be disclosed in the dissertation writing and other published manuscripts.

### Supervisory committee

The supervisory committee will monitor the data and study process from the beginning to the end. They will provide advice on the content of the study and its appropriateness with the aim of the study. Monthly meetings are planned, in which the members of the supervisory committee will be asked for further input if required. Researchers and stakeholders will be involved in the supervisory committee.

## Discussion and conclusions

The TRIMOSH study is a two-arm randomised, single-blinded, controlled, parallel trial expected to enhance the knowledge, attitude, and practice score of safety and health among food industry workers at work premises. Its design is theory-based and structured to meet the needs of this vulnerable and rapidly expanding group of workers. In this study, it is expected that no difference would be present in the characteristics of the respondents between the groups. It is hypothesised that TRIMOSH is effective in improving knowledge, attitude, and practice among food industry workers in Selangor. The results will be reported and presented in international peer-reviewed journals, conferences and other platforms. The TRIMOSH programme will be offered at the national level by the relevant authorities for the benefit of food industry workers.

## Supporting information

S1 File TRIMOSH research proposal version 3.0. (PDF)

S2 File SPIRIT_Fillable-checklist. (PDF)

## Abbreviations

DOSH: Department of Occupational Safety and Health
NIOSH: National Institute of Occupational Safety and Health
SOCSO: Social Security Organisation
TRIMOSH: Theory-Based Intervention Module on Occupational Safety and Health

## References

[1] Laws of Malaysia Act 281 Food Act. (2006). *Laws of Malaysia Act 281 Food Act 1983*. 1–24.

[2] Soon JM, Singh H, Baines R. Foodborne diseases in Malaysia: A review, Food Control 22 (2011) 823–830, doi: 10.1016/j.foodcont.2010.12.011

[3] Food Hygiene Regulation, 2009. Ministry of Health, Malaysia

[4] LAWS OF MALAYSIA, ACT 514, OCCUPATIONAL SAFETY AND HEALTH ACT 1994.

[5] DOSH. (2021). *Occupational Poisoning and Diseases Statistics 2019*. *March*, 12000. https://www.dosh.gov.my/index.php/ms/statistik/occupational-diseases-statistic/3869-2019/file

[6] DOSM. (2019). Department of statistics Malaysia: Press release annual economic statistics 2018 Food and Beverage services. *Department of Statistics Malaysia*, *March*, 1–2.

[7] U.S. DOL, BLS (2020). "Table 2—Number of cases—detailed industry level. Summary tables. 2022. Washington, DC: U.S. DOL, BLS, 2020. Apr. 3, 2022. https://www.bls.gov/iif/oshwc/osh/os/summ2_00_2020.xlsx

[8] U.S. DOL, BLS (2020). “Table R1. Number of nonfatal occupational injuries and illnesses involving days away from work by industry and selected natures of injury or illness, private industry, 2020”. Case circumstances and worker characteristics for injuries and illnesses involving days away from work by Industry. 2022. Washington, DC: U.S. DOL, BLS, 2020. Apr. 3, 2022. https://www.bls.gov/iif/oshwc/osh/case/cd_r1_2020.xlsx

[9] U.S. DOL, BLS (2020). "Table R9. Number of nonfatal occupational injuries and illnesses involving days away from work by occupation and selected natures of injury or illness, private industry, 2020". Case circumstances and worker characteristics for injuries and illnesses involving days away from work By Occupation. 2022. Washington, DC: U.S. DOL, BLS, 2020. Apr. 3, 2022. https://www.bls.gov/iif/oshwc/osh/case/cd_r9_2020.xlsx

[10] U.S. DOL, BLS (2020). "Table R4. Number of nonfatal occupational injuries and illnesses involving days away from work by industry and selected events or exposures leading to injury or illness, private industry, 2020". Case circumstances and worker characteristics for injuries and illnesses involving days away from work by Industry. 2022. Washington, DC: U.S. DOL, BLS, 2020. Apr. 3, 2022. https://www.bls.gov/iif/oshwc/osh/case/cd_r4_2020.xlsx

[11] Dapari R, Mahfot MH, Chiu Yan Yee F, Ahmad ANI, Magayndran K, Ahmad Zamzuri M‘I, et al. (2023) Prevalence of recent occupational injury and its associated factors among food industry workers in Selangor. PLoS ONE 18(11): e0293987. doi: 10.1371/journal.pone.0293987 37943862 PMC10635541

[12] Bandura A. (1997). Self-efficacy: The exercise of control. New York: Freeman.

[13] Schulz K.S., Altman D.G., Moher D. CONSORT 2010 Statement: updated guidelines for reporting parallel group randomised trials. BMJ 2010;340:c332. doi: 10.1136/bmj.c332 20332509 PMC2844940

[14] Ellison M. C. (2017). Repeated Measures Design with Generalized Linear Mixed Models for Randomized Controlled Trials, by Toshiro Tango. Journal of Biopharmaceutical Statistics, 27(6), 1121–1122. doi: 10.1080/10543406.2017.1362625 28319460

