# Supplementary material for: Theory-Based Intervention Module on Occupational Safety and Health (TRIMOSH) in improving knowledge, attitude, and practice among food industry workers: Study protocol for a randomised controlled trial
Source: PLoS One. 2024 Jan 2;19(1):e0295771. doi: 10.1371/journal.pone.0295771 (PMC10760708; doi:10.1371/journal.pone.0295771)
Supplement: S1 File — (PDF) [file pone.0295771.s001.pdf]

EFFECTIVENESS OF THEORY-BASED INTERVENTION MODULE ON OCCUPATIONAL SAFETY AND HEALTH AT FOOD PREMISE (TRIMOSH) IN IMPROVING KNOWLEDGE, ATTITUDE AND PRACTICE AMONG FOOD HANDLERS IN SELANGOR, MALAYSIA

MOHD HAFIZUDDIN BIN MAHFOT

GS62975

SV: Dr Rahmat bin Dapari

Co-SV: Dr Ahmad Iqmer Nashriq Mohd Nazan

A PROPOSAL SUBMITTED TO THE FACULTY OF MEDICINE AND HEALTH SCIENCES UNIVERSITI PUTRA MALAYSIA, IN FULFILLMENT FOR THE DOCTOR OF PUBLIC HEALTH (DrPH).

14 JULY 2022

UNIVERSITI PUTRA MALAYSIA

TABLE OF CONTENTS

EXECUTIVE SUMMARY **4**

CHAPTER 1: INTRODUCTION **6**
1.1 BACKGROUND **6**
1.2 PROBLEM STATEMENT **9**
1.3 SIGNIFICANCE OF STUDY **11**
1.4 RESEARCH QUESTION **11**
1.5 RESEARCH OBJECTIVE **12**
1.5.1 General Objective 12
1.5.2 Specific Objective 12
1.6 RESEARCH HYPOTHESIS **13**

CHAPTER 2: LITERATURE REVIEW **14**
2.1 TYPES OF OCCUPATIONAL HAZARD ENCOUNTER BY FOOD HANDLERS **14**
2.1.1 Physical Hazard 14
2.1.2 Chemical Hazard 15
2.1.3 Biological Hazard 16
2.1.4 Mechanical Hazard 16
2.1.5 Psychosocial Hazard 17
2.2 KNOWLEDGE, ATTITUDE, AND PRACTICE (KAP) ON OCCUPATIONAL SAFETY AND HEALTH AMONG FOOD HANDLER **17**
2.3 FACTORS ASSOCIATED WITH KNOWLEDGE, ATTITUDE AND PRACTICE AMONG FOOD HANDLERS **18**
2.3.1 Effectiveness of Intervention Module in Improving KAP On OSH Among Food Handlers 23
2.4 THEORETICAL FRAMEWORK TO DEVELOP THEORY-BASED INTERVENTION MODULE ON OCCUPATIONAL SAFETY AND HEALTH AT FOOD PREMISE **23**
2.4.1 The Health Belief Model 23
2.4.3 The Tone's Health Action Model 25
2.4.1 Social Cognitive Theory 27
2.4.1 Knowledge, Attitude and Practice (KAP) Theory 28
2.5 CONCEPTUAL FRAMEWORK 29

CHAPTER 3: METHODOLOGY **31**
3.1 STUDY LOCATION **31**
3.2 STUDY DESIGN **32**
3.2 STUDY POPULATION **32**
3.3 SAMPLING POPULATION **33**
3.3.1 Sampling unit 33
3.3.2 Inclusion criteria 33
3.3.3 Exclusion criteria 33

3.4 SAMPLING FRAME **33**
3.5 SAMPLING TECHNIQUE **34**
3.6 SAMPLE SIZE **35**
3.6 VARIABLES **37**
3.6.1 Dependent Variable 37
3.6.2 Independent Variables 37
3.7 INSTRUMENT **38**
3.8 DEVELOPMENT OF INTERVENTION MODULE **39**
3.9 DATA COLLECTION METHODS **40**
3.10 DATA ANALYSIS **40**
3.11 QUALITY CONTROL **41**
3.12 ETHICAL APPROVAL **41**
3.13 OPERATIONAL DEFINITION OF VARIABLES **42**
3.13.1 Dependent Variable 42
3.13.2 Independent Variables 42

CHAPTER 4: RESULTS **44**
4.1 RESULTS DUMMY TABLE **44**

ACKNOWLEDGEMENTS **48**
REFERENCES **49**
APPENDICES **53**
Budget Planning 53
Gantt Chart 54
Information Sheet and Informed Consent Form (English) 55
Information Sheet and Informed Consent Form (Malay) 57
Intervention Module Outline 59
Questionnaire 66

EXECUTIVE SUMMARY

Abstract of thesis presented to the Senate of Universiti Putra Malaysia in fulfilment of the requirement for the degree of Doctor of Public Health

EFFECTIVENESS OF THEORY-BASED INTERVENTION MODULE ON OCCUPATIONAL SAFETY AND HEALTH AT FOOD PREMISE (TRIMOSH) IN IMPROVING KNOWLEDGE, ATTITUDE AND PRACTICE AMONG FOOD HANDLERS IN SELANGOR, MALAYSIA

By

MOHD HAFIZUDDIN BIN MAHFOT

May 2022

Chair : Dr. Rahmat bin Dapari
Faculty : Faculty of Medicine and Health Sciences

Introduction:

The food and beverages industry contributed a significant income to Malaysia's Gross Domestic Products (GDP). The contribution is projected to swell more in the upcoming years. As the industry expands, the demand for the workforce in the food premises will also continuously increase. The workforce substantially involves workers from the food processing sector, food distribution sector, and food service sector in the food premises. Regardless of the section, it is essential for food handlers who work in food premises to be equipped with the proper knowledge, attitude, and practice (KAP) towards safety and health. This is because they are exposed to risks arising from physical, chemical, biological, mechanical, and social hazards while performing their duties like in any other occupation. Therefore, a well-trained food handler equipped with the right KAP for safety and health will contribute to a safer and positive working environment, smoother work operation, better services, reduced accidents at the workplace, and subsequently increased workers' motivation and productivity.

Objective:

The general objective of this study is to develop, implement, and evaluate the effectiveness of the occupational safety and health training program in increasing the KAP among food handlers.

Methodology:

The study is a single-blinded, cluster randomized controlled trial study, expected to be conducted from 1st December 2023 to 30th November 2024 among 139 registered food handlers in Selangor under municipal in the working-age between 18-65 years old. The respondets will be allocated into intervention and control group. The intervention group will reveive TRIMOSH intervention while the control group will receive standard training. The data will be collected via self-administered questionnaires and has been analyzed using SPSS version 25.

Result:

A total of 139 participants expected to be finally selected to participate in the study with response rate of more than 80%. At baseline, the characeteristics of the sociodemographic, secioeconomic, working experience and training attendence of food handlers hypothesized to show no difference in between group. It is hypothesized that there will be a significant improvement on knowledge, attitude and practice (KAP) on occopational safety and health at food premises among food handlers in the intervention group compared to the control post training and 1 month follow up. In the GLMM analysis, it is hypothesized that the TRIM-OSH is associated with improving KAP among food handlers on occupational safety and health at food premises.

Conclusion:

In order to achieve the desired outcome, there is a role of structured training. Thus, a theory-based intervention module on occupational safety and health at food premises (TRIM-OSH) to improve KAP on occupational safety and health among food handlers will be developed and examined through an intervention study. In this study, the participants will be randomly selected and subsequently assessed for their KAP pre and post-participation in the module. In addition, the study will assess the module's effectiveness in improving KAP of occupational safety and health among the targeted population in Selangor, through statistical analysis, with the aim of national implementation in the future.

CHAPTER 1

INTRODUCTION

1.1 BACKGROUND

1.1.1 Industry Overview

The food and beverages industry contributed a significant income to Malaysia’s Gross Domestic Products (GDP). Its contribution is projected to swell more in the upcoming years. The food and beverages industry can be divided into three main sections: food processing, food distribution, and food services. Food processing sectors mainly involve edible products and preparations such as poultry, seafood, cereal, etc. The food distribution sector refers to the supply and distribution of local-produced and imported products to the end-user through small-scale retailers such as local grocery stores or large-scale retailers such as Tesco, Jaya Grocer, AEON Big, etc. Food service sector includes those businesses involved with fine dining restaurants, international chains, hotel, pub, bars, cafes, and stalls businesses.

The Malaysian Investment Development Authority (MIDA) data shows that the food-processing sector alone accounts for about 10% of Malaysia’s manufacturing output. Processed food contributed about RM21.76 billion and is exported to more than 200 countries, while the import value of processed food amounted to RM20.27 billion in 2019. The agency also estimated that the annual growth rate of the Food and Beverages industry stands at 7.6% in 2019. (MIDA, 2019). In 2017, Malaysia's food distribution sectors value was estimated at RM20.48 billion (FIT, 2020). In a similar year, the food services sector’s gross output in Malaysia was valued at RM82.8 billion, as reported by the Department of Statistics Malaysia (DOSM) in their report (DOSM, 2019). The Covid-19 pandemic has undoubtedly dampened the growth of the food industry in the current year; however, the economic situation is expected to recover as the pandemic settles down.

Based on the data by Economic Planning Unit, Prime Minister's Department (EPU), the vast number of workforces in the food and beverages industry can be found under class C (Manufacturing industry), class G (Wholesale and retail trade, repair of motor vehicles and motorcycles) and class I (Accommodation and food service activities). The numbers of employed persons in all mentioned classes of Industry show a consistently increasing trend from year to year since 2010 till the year the Covid-19 crisis sets in. In 2019, the number of employed persons in class C was estimated at 2.7 million, class G at 2.6 million, and class I at 1.5 million, which comprised up to 45% of employment in comparison with other industries such as class A (Agriculture, forestry, and fishing), class F (Construction), class P (Education) and, etc. (EPU, 2019).

As the Covid-19 pandemic crisis became pandemic, the economy recovered, and the industry expanded, the demand for the workforce in the food and beverages industry at food premises is expected to increase. Food premises are the premises used for or in connection with the preparation, preservation, packaging, storage, conveyance, distribution, or sale of any food or the relabelling, reprocessing, or reconditioning of any food (Laws of Malaysia Act 281 Food Act, 2006). As the workers' volume increases, the number of accidental injuries or occupational diseases related to the food and beverages industry is expected.

1.1.2 Injuries and illnesses in the Industry

As in any other profession, an individual who works in food premises will be exposed to various hazards related to the nature of their work. Failure to ensure occupational safety and health at food premises may lead to accidental injury, or occupational diseases that are not only detrimental to the food handler's general well-being but also affect the productivity of the organization as the affected workers or employees may have to stay days away, restricted, or transferred (DART) from work.

Based on the Bureau of Labor Statistics data, United States Department of Labor (BLS), the highest number of nonfatal occupational injuries and illnesses involving days away from work (DAFW) in private Industry in 2020 goes to those who involve in health care and social assistance. The observation was tallied with the Covid-19 pandemic situation and contributed to about 30.4% of total reported cases of 1.18 million. It is followed by manufacturing (14.1%), retail trade (12.8%), transportation and warehousing (7.8%), and accommodation and food services (7.2%). The mentioned industries already contributed more than 70% of the reported cases (U.S. DOL, BLS (a), 2020). However, the number of food handlers working at food premises in this data can be found in the manufacturing, retail trade, accommodation, and food services sector. In a further analysis based on occupation, the highest number of nonfatal occupational injuries and illnesses involving days away from work (DAFW) in private Industry involving food handlers within the industries contribute about 22% of total reported cases or around 0.3 million cases (U.S. DOL, BLS (b), 2020).

Of all the reported injuries and illnesses cases, cuts, lacerations and punctures (15%) was the highest contributors of injuries and illnesses for known classified cases among food handlers, followed by sprain, strains, and tears (15%), soreness and pain (13%), thermal burn (8%), bruises and contusions (5%), fractures (4%), multiple traumatic injuries (1%), chemical burns and corrosions (1%), carpal tunnel syndrome (1%), amputation (0.4%) and tendonitis (0.06%). However, there was a sum of reported cases that were non-classifiable and contributed to about 35% of the total reported cases among food handlers (U.S. DOL, BLS (c), 2020). The highest number of injuries and illnesses experienced by food handlers have resulted from exposure to harmful substances or environments (34%), followed by contact with objects or equipment (22%), falls, slips, and trips (21%), overexertion and bodily reaction (19%), transportation incidents (2%), violence and other injuries by persons or animal (1%), other events or exposures (0.4%) and fires and explosions (0.09%) (U.S. DOL, BLS (d), 2020).

From the similar data, the top ten occupations that have been reported to have the highest incidence of occupational injuries and illnesses among food handlers were observed among those who work in food preparation and serving related occupations (25%), cooks, and food preparation workers (10%), food and beverage serving workers (9%), food processing workers (6%), cooks (5%) fast food and counter workers (5%), food preparation workers (5%), supervisors of food preparation and serving workers (4%) butchers and other meat, poultry and fish processing workers (3%) and other food preparation and serving related workers (3%) (U.S. DOL, BLS (c), 2020).

1.2 PROBLEM STATEMENT

Since 1996, the Ministry of Health Malaysia (MOH) has launched the food handler training course to give exposure and awareness to food handlers on food safety, hygiene, self-hygiene, and premise hygiene, mainly to prevent food poisoning incidents in the country. Subsequently, under Food Hygiene Regulations 2009, all food handlers are compulsory to attend the Food Handler Training Programme organized by any school or institute that has been certified by MOH. Food handlers are defined as individuals who are (1) directly involved in the food preparation, (2) come into contact with food or food contact surfaces, and (3) handle packaged or unpackaged food, or appliances, on any food premises (Laws of Malaysia Act 281 Food Act, 2006). Under this regulation, any food handler who fails to undergo training or obtain a Certificate of Food Handlers Training shall be liable to a fine or compound not exceeding RM10,000 or imprisonment for a term not exceeding 2 years (Food Hygiene Regulation, 2009).

The food handler training module by MOH has been formalized and uniformed and comprises four sections: introduction, food hygiene, food safety, and critical factors of food poisoning (MOH, 2014). However, despite this module being compulsory, the data reported by the Department of Occupational Safety and Health (DOSH)

shows that the number of cases reported and cases confirmed for occupational poisoning and disease generally show a consistently increasing trend from 2010 to 2019.

Regarding occupational safety and health of workers who work as food handlers in Malaysia, they are generally governed and regulated by Act 514 Occupational Safety and Health (OSHA) Act 1994 as for any other occupations. It is within the spirit of the act to secure the safety, health, and welfare of persons at work to protect the workers and others from any risk or health implication associated with the activities of persons at work and prevent occupational diseases. An occupational disease is defined as any disease contracted due to exposure to risk factors arising from work activity (DOSH, 2021). However, there is currently no occupational safety and health training module, required or made compulsory by the government for food handlers to equip them with appropriate knowledge, attitude, and practice on occupational safety and health to prevent injuries and illnesses related to their work.

Based on local statistics, the manufacturing industry, including the food and beverages sector, accounted for the most significant contributor to all confirmed occupational poisoning and disease cases at 82.3% (6792). On the other hand, the number of confirmed cases from hotels and restaurants and wholesale and retail trades accounted for 0.5% (41) and 0.3% (25) of total confirmed cases. According to the sector, in terms of accidental injury at the workplace, DOSH Malaysia generally reported an increasing trend of accidents among workers from year to year. In 2020 only, 62% (4202) of accidents with temporary disability, 84% (231) of accidents with a permanent disability, and 34% (73) of death reported to DOSH were coming from the manufacturing industry. On the other hand, 2% (137) of accidents with temporary disability, 0.4% (1) of accidents with a permanent disability, and 0.7% (2) of death reported to DOSH came from the hotel and restaurants industry. Wholesale and retail trade reported a 2% (126) of accidents

with temporary disability, 0.4% (1) of accidents with a permanent disability, and 0.4% (1) of death to DOSH.

Based on the available data on occupational poisoning, occupational diseases, and accidents that lead to temporary and permanent disability among workers, especially in the food and beverages industry, related training is appropriate for food handlers to prevent such occurrences.

1.3 SIGNIFICANCE OF STUDY

The study will be able to provide a more in-depth understanding to the researcher, educator, and trainer who are involved in the food industry on the issue of occupational safety and health among food handlers and able to help them in identifying the training content that is suitable, related and appropriate to the food industry trainee.

The analysis of this study will allow the researcher to identify the effectiveness of the developed theory-based intervention module on occupational safety and health at food premises (TRIM-OSH) in improving KAP on the occupational safety and health among the food handlers working on food premises. It will also be able to guide the researcher on the feasibility of this program on a larger scale, e.g., state or national level.

A practical theory-based intervention module on occupational safety and health at food premises (TRIM-OSH) may help the government add value to the current food handler training program by including the occupational safety and health component to prevent occupational-related poisoning, diseases, and accidents at food premises.

1.4 RESEARCH QUESTION

Why should this occupational safety and health training program be done among food handlers? Is there any deficiency tied to the need?

Are this occupational safety and health training program effective in increasing the knowledge, attitude, and practice among food handlers regarding occupational safety and health at work?

1.5 RESEARCH OBJECTIVE

1.5.1 GENERAL OBJECTIVE

The general objective of this study is to develop, implement, and evaluate the effectiveness of the occupational safety and health training program in increasing the KAP among food handlers.

1.5.2 SPECIFIC OBJECTIVE

The specific objectives of this study are:

1. To describe the baseline sociodemographic, socioeconomic, working experience, training attendance, and means KAP score of food handlers.

2. To develop and implement a theory-based intervention module for food handlers in Selangor, Malaysia.

3. To evaluate the effectiveness of the SCT constructs as the main framework of the intervention module in improving KAP among food handlers between the intervention and control group.

4. To describe and compare the effectiveness of the TRIM-OSH on the change of knowledge, attitude and practice compared to standard training among food handlers between and within groups at the end, and one-month post training, after controlling for covariates.

1.6 RESEARCH HYPOTHESIS

The research hypothesis of this study is:

H1: The sociodemographic, socioeconomic, working experience, and training attendance characteristics of the intervention and control groups would be the same.

H01: There is no significant differences in the sociodemographic, socioeconomic, working experience, and training attendance characteristics of the intervention and control groups.

H2: The KAP on occupational safety and health at food premises of the intervention and control groups at the baseline would be the same.

H02: There is no significant differences in the KAP on occupational safety and health at food premises of the intervention and control groups at the baseline.

H3: The SCT constructs as the main framework of the intervention module is effective in improving KAP among food handlers between the intervention and control group.

H03: The SCT constructs as the main framework of the intervention module have no significant effect in KAP among food handlers between the intervention and control group.

H4: The TRIM-OSH effectively improves KAP on occupational safety and health at food premises among food handlers.

H04: There is no significant differences in the KAP on occupational safety and health at food premises of the intervention and control groups at post-intervention.

CHAPTER 2

LITERATURE REVIEW

2.1 TYPES OF OCCUPATIONAL HAZARDS ENCOUNTERED BY FOOD HANDLERS

Occupational hazards can be present in any workplace. However, depending on the type of food premises they are working, food handlers may be exposed to various hazards that could be detrimental to their safety. To describe the characteristics, hazards at food premises can be classified into physical, chemical, biological, mechanical, and social hazards (Petricciani, 2009).

2.1.1 Physical Hazard

Physical hazards can be defined as factors within the environment that can harm the body with or without direct contact. For example, a food handler involved in processing raw materials such as chicken, seafood, and meat may be exposed to physical hazards due to low temperatures. They might have to work for a long hour in a refrigerated room to prevent the product from spoiling. However, this will expose the workers to more prone to frostbite, respiratory disorders, and rheumatic disorders (Thetkathuek et al., 2015). In contrast, a food handler might work under high temperatures, mainly when allocated near a cooking range. Without a proper ventilation system, the heat produced during the cooking process might cause excessive sweat production, leading to acute dehydration, heat exhaustion, heat cramps, heatstroke, and burns (Zulkarnain et al., 2020).

Another example of physical hazards commonly found at food premises is noise. This is especially common in the food processing industry that involves the use of machines in their processing line, for example, for mixing and grinding purposes, in a factory setting. However, the food handlers who work in the kitchen in hotels,

restaurants, and franchise food chains can also be exposed to the hazard of noise that may come from an exhaust fan, food processor, blender, grinder, etc. Prolonged exposure to excessive noise may lead to non-auditory effects such as anxiety, lethargy, reduced productivity, and increased agitation, besides the much-avoided auditory effects of hearing loss (Mirza et al., 2018). Furthermore, mixing and grinding in the food industry, such as grinding and mixing grain, beans, nuts, and herbs, can also lead to the suspension of dust in the air. This will expose the food handlers to inhalation of dust particles, especially likely to result in respiratory disorders such as Baker’s lung disease and allergies (De Matteis et al., 2017).

In the food processing, distribution, or food service segment of the food and beverages industry, food handlers commonly have to perform their work by standing for long hours. In addition, there are also involved in transferring food items that carry a substantial amount of weight at times. Without proper posture and technique, especially during lifting up and putting down a heavy load, can cause recurrent musculoskeletal symptoms such as upper and lower back pain, back and thigh muscle sprain and strain, and prolapsed intervertebral disc in a more severe condition (Bovenzi et al., 2017). In other situations, the working area might have been designed without consideration of the ergonomics of the food handlers. For example, an excessive discrepancy in the height of a working table and the sitting chair of the food handlers may easily cause back discomfort and fatigue.

2.1.2 Chemical Hazard

A chemical hazard can be defined as any chemical preparation in the workplace in any form (solid, liquid, or gas). Besides being involved in the preparation, cooking, and serving of food, food handlers also involve cleaning the customers’ table, kitchen, utensils, etc., where they come in contact with the chemical solution in the form of detergents or disinfectants. The safety level of solutions differs from one to another. Most products must comply with specific safety standards before

entering the market for general use. However, the individual response among food handlers towards the chemical solution might also differ. During the cleaning process, prolonged exposure to direct contact with the chemical solution, such as cleaning products or solvents, can cause skin irritation, occupational contact dermatitis, or respiratory problems.

2.1.3 Biological Hazard

A biological hazard can be defined as any hazard associated with working with animals, people, or infectious plant materials. In the context of occupational safety and health, food handlers can be carriers of an infective organism and become the index source of infection to other co-workers at the food premises. For example, in the Covid-19 pandemic period, one can easily get infected if the standard operating procedures and guidelines for preventing Covid-19 infection are not followed, such as mask-wearing, hand hygiene, and social distancing. Besides that, food handlers may also come in contact with other common infective agents for foodborne pathogens such as *Bacillus cereus, Campylobacter jejuni, Clostridium botulinum, Clostridium perfringens, Cronobacter sakazakii, Esherichia coli, Listeria monocytogenes, Salmonella spp., Shigella spp., Staphylococcus aureus, Vibrio spp. and Yersinia enterocolitica), viruses (Hepatitis A and Noroviruses)* and *parasites (Cyclospora cayetanensis, Toxoplasma gondii* and *Trichinella spiralis* (Bintsis, 2017). Food handlers who are at active infection, or carriers, can transfer these pathogens into the food and compromise their health and food safety if good hand hygiene is not in practice.

2.1.4 Mechanical Hazard

A mechanical hazard can be defined as any hazard associated with the moving parts of a machine or work equipment. For example, a food handler working at the

meat processing branch often uses sharp and dangerous hand tools. During the initial part of the raw materials process, sharp and heavy butcher's knives are routinely used to cut and trim the meat by hand. As the working area where the cutting and trimming process can get wet and slippery, the proper boot can reduce the risk of slipping and falling. One must also be skilled in handling the knives. A food handler might have to deal with a slaughter machine, meat cutting machine, or meat mincer machine at another segment. The machine must be dealt with high safety precautions as improper handling might cause severe physical injuries leading to temporary or permanent disabilities or even death (Tomoda & International Labour Office. Industrial Activities Branch., 1993).

2.1.5 Psychosocial Hazard

Psychosocial hazards can be defined as stressors that cause stress (short-term effects) and strain (long-term effects). These hazards are related to issues at the workplace such as workload, lack of control, respect, etc. Food handlers are commonly perceived as low-skilled occupations that do not require high-order thinking skills. At food premises, it is not uncommon to see that some workers work at the same level for years as there is little opportunity for career progression. Most of them work in the unorganized sector. The food handlers are also often exploited with low wages that are incomparable to their actual skills, strenuous activity, and restricted leave. The stress they have to endure regarding their duty might toll their mental and physical health (Dudeja & Singh, 2017).

2.2 KNOWLEDGE, ATTITUDE, AND PRACTICE (KAP) ON OCCUPATIONAL SAFETY AND HEALTH AMONG FOOD HANDLERS

As food handlers will be exposed to various types of hazards, all food handlers must be equipped with good KAP to prevent occupational-related injury and

diseases among food handlers working in the food and beverages industry. The food handlers equipped with good knowledge of occupational safety and health would allow them to perform their duty safely. Good knowledge will encourage the workers always to put safety and health as the main priority, creating a safe climate at work. Positive attitudes towards occupational safety and health-related activities can be seen, for example, via active participation among workers in safety and health training organized by the organization and their compliance with safety and health-related policy and regulations at work and vice versa. Excellent practice, action, or behavior towards occupational safety and health at work may reduce the risk of accidents. Exploring KAP of workers toward occupational safety and health can be used to aid in evidence-based intervention, which can improve work situations or the target behavior (Goh & Chua, 2016).

2.3 FACTORS ASSOCIATED WITH KNOWLEDGE, ATTITUDE, AND PRACTICE AMONG FOOD HANDLERS

In the literature review, there were limited studies found under the specific theme of occupational safety and health among food handlers at food premises. The literature commonly ends up with a study that look into the theme of food safety and food hygiene rather than food handler's safety and food handler's health. However, upon revising the training module on food safety and food hygiene of few agencies such as MOH, there were also component of occupational safety and health in the module that been taught to food handlers, but from the point of view of food safety and food hygiene such as hand washing practices (MOH, 2014). From food safety and food hygiene point of view, hand washing should be practiced by food handlers to prevent food contamination. However, from the occupational safety and health point of view, the objective of hand washing practices is to protect the food handlers from getting themselves infected for example, by infectious animals or plants.

Due to the limitation and the common similarities found in the training module of food safety and food hygiene, literature review on factors associated with KAP on

occupational safety and health at food premises will also include such study and presented in the table as shown below. The factors found can be categorized into sociodemographic, socioeconomic, working experience and previous training:

Variable	Reference	Study Details	Findings	p value
Age	(Nee & Sani, 2011)	Assessment of Knowledge, Attitudes and Practices (KAP) Among Food Handlers at Residential Colleges and Canteen Regarding Food Safety.	There was no statistical difference between the knowledge aspects and the age groups.	p>0.05
Age	(Nee & Sani, 2011)	Assessment of Knowledge, Attitudes and Practices (KAP) Among Food Handlers at Residential Colleges and Canteen Regarding Food Safety.	There was no statistical difference between the attitude aspects and the age groups.	p>0.05
Age	(Nee & Sani, 2011)	Assessment of Knowledge, Attitudes and Practices (KAP) Among Food Handlers at Residential Colleges and Canteen Regarding Food Safety.	There was no statistical difference between the practice aspects and the age groups.	p>0.05
Gender	(Nee & Sani, 2011)	Assessment of Knowledge, Attitudes and Practices (KAP) Among Food Handlers at Residential Colleges and Canteen Regarding Food Safety.	There was no significant difference in average points of knowledge with respect to the gender variable.	p>0.05
Gender	(Nee & Sani, 2011)	Assessment of Knowledge, Attitudes	There was no significant different	p>0.05

		and Practices (KAP) Among Food Handlers at Residential Colleges and Canteen Regarding Food Safety.	in average points of attitude with respect to the gender variable.	
Gender	(Nee & Sani, 2011)	Assessment of Knowledge, Attitudes and Practices (KAP) Among Food Handlers at Residential Colleges and Canteen Regarding Food Safety.	There was significant different in average points of practice with respect to the gender variable.	$p<0.05$
Level of Education	(Alemayehu et al., 2021)	Food Safety Knowledge, Handling Practices and Associated Factors Among Food Handlers Working in Food Establishments in Debre Markos Town, Northwest Ethiopia, 2020: Institution-Based Cross-Sectional Study	Food handlers who did not attain any formal education were 77.0% less likely to have good food safety knowledge as compared to those food handlers who had completed secondary educational level.	$P<0.001$
Working experience	(Nee & Sani, 2011)	Assessment of Knowledge, Attitudes and Practices (KAP) Among Food Handlers at Residential Colleges and Canteen Regarding Food Safety.	There was a significant difference between the knowledge aspect and the duration of working experiences.	$p<0.05$
Working experience	(Nee & Sani, 2011)	Assessment of Knowledge, Attitudes and Practices (KAP) Among Food Handlers at Residential Colleges	There was no significant difference between the attitude aspect and the duration of working experiences.	$p>0.05$

		and Canteen Regarding Food Safety.		
Training attendance	(Nee & Sani, 2011)	Assessment of Knowledge, Attitudes and Practices (KAP) Among Food Handlers at Residential Colleges and Canteen Regarding Food Safety.	There was no significant difference between knowledge and attendance to training course.	$p>0.05$
Training attendance	(Has et al., 2018)	An Assessment on Pre- and Post-Food Hygiene Training on Food Safety's KAP Level Among Food Handlers In Kuala Terengganu And Kuala Nerus	There was a significant difference in knowledge scores pre- and post-food hygiene training.	$p<0.05$
Training attendance	(Nee & Sani, 2011)	Assessment of Knowledge, Attitudes and Practices (KAP) Among Food Handlers at Residential Colleges and Canteen Regarding Food Safety.	There was no significant difference between practice and attendance to training course.	$p>0.05$
Training attendance	(Nee & Sani, 2011)	Assessment of Knowledge, Attitudes and Practices (KAP) Among Food Handlers at Residential Colleges and Canteen Regarding Food Safety.	There was significant difference between attitude and attendance to training course.	$P<0.05$
Training attendance	(Has et al., 2018)	An Assessment on Pre- and Post-Food Hygiene Training on Food Safety's KAP Level Among Food	There was no significant difference in attitudes scores pre- and post-food hygiene training.	$p>0.05$

		Handlers In Kuala Terengganu And Kuala Nerus		
Training attendance	(Has et al., 2018)	An Assessment on Pre- and Post-Food Hygiene Training on Food Safety's KAP Level Among Food Handlers In Kuala Terengganu And Kuala Nerus	There was a statistically significant difference in practice scores pre- and post-food hygiene training.	$p<0.05$
Supervisor practices (Active)	(Mohd Ishanuddin et al., 2019)	An Investigation of Knowledge, Attitude and Practice of Occupational Safety and Health (OSH) on Safety Climate at Workplace in Manufacturing Industry	Knowledge and active practices factors showed positive moderate correlation.	$p<0.01$
Supervisor practices (Proactive)	(Mohd Ishanuddin et al., 2019)	An Investigation of Knowledge, Attitude and Practice of Occupational Safety and Health (OSH) on Safety Climate at Workplace in Manufacturing Industry	Knowledge and proactive practices factors showed positive moderate correlation.	$p<0.01$
Supervisor practices (Declarative)	(Mohd Ishanuddin et al., 2019)	An Investigation of Knowledge, Attitude and Practice of Occupational Safety and Health (OSH) on Safety Climate at Workplace in Manufacturing Industry	Knowledge and declarative practices factors have a strong positive correlation.	$p<0.01$

2.3.1 EFFECTIVENESS OF INTERVENTION MODULE IN IMPROVING KAP ON OSH AMONG FOOD HANDLERS

There were also limited studies that specifically look into the effectiveness of intervention module in improving KAP on occupational safety and health among food handlers. There were more studies that look into the effectiveness of an intervention module in improving KAP on food safety and food hygiene among food handlers. Thus, due to the limitation the literature review on the effectiveness of intervention module in improving KAP on occupational safety and health among food handlers also include study that examine the effectiveness of an intervention module in improving KAP on food safety and food hygiene among food handlers. The summary of the findings is tabulated in the table below:

Variable	Reference	Study Details	Findings	p value
Knowledge	(Adamu et al., 2021)	Impact of An Intervention Program on Improvement of Knowledge, Attitudes, Practices (KAP) On Food and Occupational Health Safety Among Cattle Abattoir Workers in Malaysia.	Comparison of score in pre and post- intervention phases indicated a significant difference in knowledge.	$p<0.0001$
Knowledge	(Isara & Isah, 2009)	Knowledge and practice of food hygiene and safety among food handlers in fast food restaurants in Benin City, Edo State	Knowledge was significantly influenced by previous training in food hygiene and safety.	$p = 0.002$
Knowledge	(Mohd Ishanuddin et al., 2019)	An Investigation of Knowledge, Attitude and Practice of Occupational Safety and Health (OSH) on Safety Climate at Workplace in Manufacturing Industry	If knowledge of workers about safety is high, the workers perception towards supervisor’s commitment in relation to comply with safety and health regulation is also high.	$p < 0.01$

Knowledge	(Has et al., 2018)	An Assessment on Pre- and Post-Food Hygiene Training on Food Safety's KAP Level Among Food Handlers In Kuala Terengganu And Kuala Nerus	There was a significant difference in knowledge scores pre- and post-food hygiene training.	$p<0.05$
Attitude	(Adamu et al., 2021)	Impact of An Intervention Program on Improvement of Knowledge, Attitudes, Practices (KAP) On Food and Occupational Health Safety Among Cattle Abattoir Workers in Malaysia.	Comparison of score in pre and post- intervention phases indicated a significant difference in attitude (0.025) but no significant difference was observed in practice scores (p=0.115) based	$p =0.025$
Attitude	(Mohd Ishanuddin et al., 2019)	An Investigation of Knowledge, Attitude and Practice of Occupational Safety and Health (OSH) on Safety Climate at Workplace in Manufacturing Industry	The attitude on safety at the workplace have no significant correlation with practices.	$p=0.616$
Knowledge	(Has et al., 2018)	An Assessment on Pre- and Post-Food Hygiene Training on Food Safety's KAP Level Among Food Handlers in Kuala Terengganu And Kuala Nerus	There was no significant difference in attitudes scores pre- and post-food hygiene training.	$p>0.05$
Practice	(Adamu et al., 2021)	Impact of An Intervention Program on Improvement of Knowledge, Attitudes, Practices (KAP) On Food and Occupational Health	Comparison of score in pre and post- intervention phases indicated no significant difference was	$p=0.115$

		Safety Among Cattle Abattoir Workers in Malaysia.	observed in practice scores.	
Practice	(Isara & Isah, 2009)	Knowledge and practice of food hygiene and safety among food handlers in fast food restaurants in Benin City, Edo State	Food handlers who had worked for longer years in the fast food restaurants had better practice of food hygiene and safety.	$p = 0.036$
Practice	(Mohd Ishanuddin et al., 2019)	An Investigation of Knowledge, Attitude and Practice of Occupational Safety and Health (OSH) on Safety Climate at Workplace in Manufacturing Industry	For the workers that acquired good practice of safety at the workplace, increased in the aspect of practicing safety would directly increase the perception of workers on supervisor declarative practices on the safety and health policies implemented at work.	$p < 0.01$

2.4 THEORETICAL FRAMEWORK TO DEVELOP THEORY-BASED INTERVENTION MODULE ON OCCUPATIONAL SAFETY AND HEALTH AT FOOD PREMISE (TRIM-OSH)

There are numerous social cognitive theories available in health evaluation and behavioral prediction. Most theories discussed on how we could determine various related behaviors through the theory structure. However, the application of some theory or model is more suitable than the other in determining specific interventions

to modify such behaviors so that the desired behaviors can be predicted and performed (Clayton & Griffith, 2008). Thus, it is essential to understand the theoretical knowledge of particular behavior and behavioral change so that the potential efficacy of the developed intervention can be maximized. In essence, the theory will impart known knowledge on how individuals act and what are the moderators that may contribute to their actions, based on the nature of human behavior and other surrounding influences. In this research study, the application of theory being instilled as an integral part of the development of the intervention module, such as being recommended by the UK Medical Research Council's guidance for developing and evaluating complex interventions (Campbell et al., 2000).

2.4.1 The Health Belief Model

The Health Belief Model (HBM) is one of the most extensively used models of frameworks in the scientific research of health behavior. HBM provides a comprehensive understanding in explaining change and maintainence of desired health-related behaviors and a guiding framework for health behavior interventions. HBM consists of conceptual constructs that suggest why an individual comes to a point of action, for example, to prevent certain situations. The construct includes perceived susceptibility, which can be defined as belief about the likelihood of getting a disease or condition and becoming a threat, perceived severity, which can be defined as feelings about the seriousness of getting a disease or condition and becoming a threat if nothing was done, perceived benefits, which can be defined as an individual perceived benefits towards various action for diminishing the threat of the disease or condition, perceived barrier, which can be defined as the potential negative aspects of a particular action or practice, and self-efficacy, which defined as “the conviction that one can successfully execute the behavior required to produce the outcomes” (Bandura, 1997). HBM identified that besides sociodemographic and socioeconomic factors,

knowledge is an important modifying factor that influences individuals' beliefs and, subsequently, behaviors such as been depicted in the figure below.

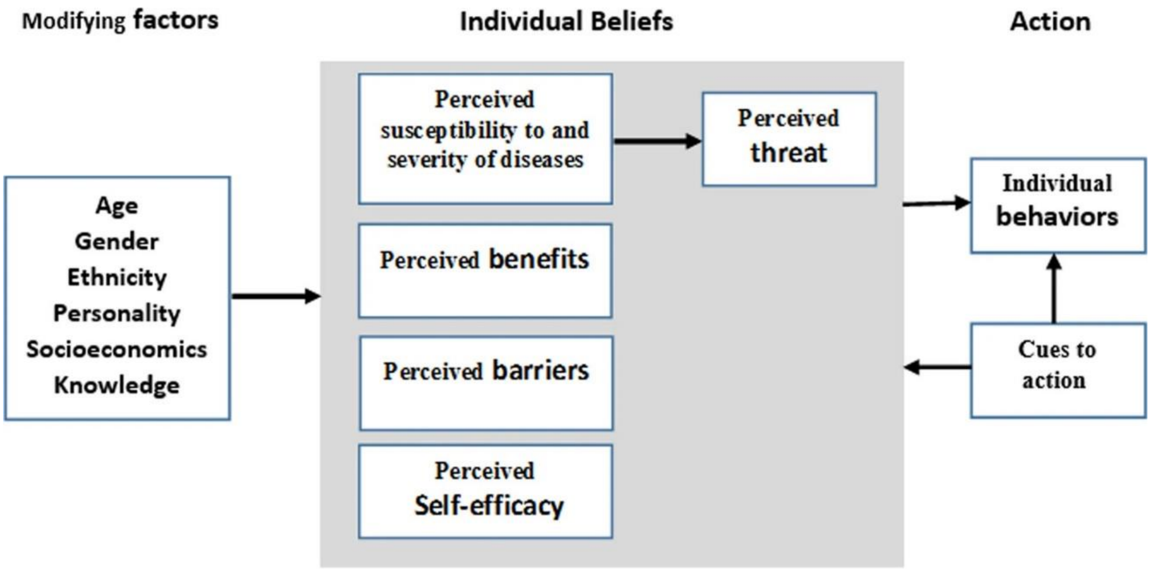

Figure 1. The Health Belieff Model

Source: Adapted from Glanz, K., Rimer, B. K., & Viswanath, K. (Eds.). (2015)

2.4.2 The Theory of Reasoned Action

Theory of Reasoned Action (TRA) is one of the most commonly used theories of behavior and behavior changes across the social and behavioral sciences (Davis et. al., 2015). Based on TRA model, an individual decision to engage in specific desired behavior is determined by their attitude and social normative perceptions towards the behavior. In further elaboration, attitude can be defined as an individual's belief about the outcome of certain behavior. An individual with strong beliefs that positively valued outcome is more likely to perform the desired behavior and being referred to have a positive attitude towards the behavior. On the other hand, those with strong beliefs that negatively value outcome is less likely to perform the behavior and are said to have a negative attitude towards the behavior. As for subjective norm, it can be defined as the individual normative belief towards the outcome of certain behavior. Similarly, an individual who believes that certain

referents think they should perform a behavior and is motivated to meet the expectations of those referents will hold a positive subjective norm and vice versa.

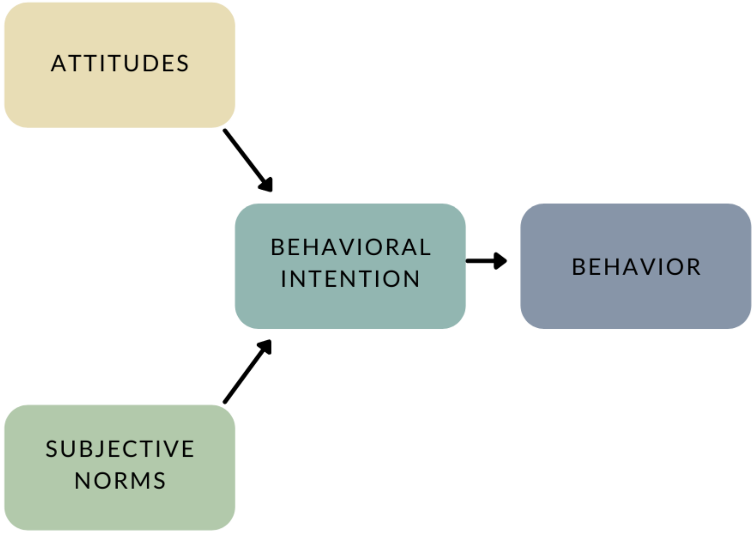

Figure 2. Theory of Reasoned Action
Source: Adapted from Fishbein, M. & Ajzen, I. (1980)

2.4.3 The Tone's Health Action Model

The Tones' Health Action Model originated from the two models in health evaluations and behavioral predictions mentioned above, the Health Belief Model and the Theory of Reasoned Action (Frizelle, 1995).

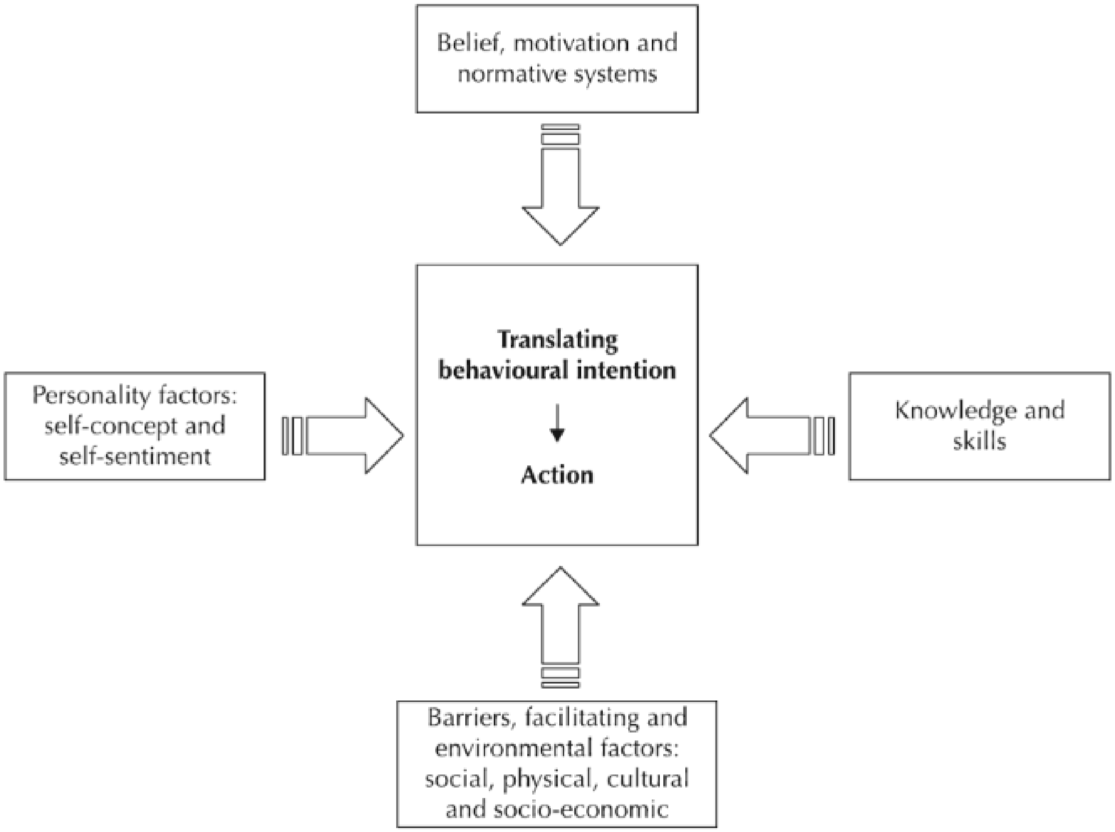

Figure 3. Health Action Model

Source: Adapted from Goodman-Brown and Gottwald (2008) and Tones and Tilford (2001)

Based on this model, the normative system is considered central to all behavior, distinguishing between attitudes and beliefs, behavioral intentions, and resultant actions. Beliefs and attitudes may interact to produce a behavioral intention. The intention leads to an advocated action when appropriate social and environmental conditions prevail (Frizelle, 1995). Other influencing factors are also incorporated, for example, the knowledge about food hygiene obtained from a food hygiene course and the influence of norms, which could be influenced by the provision of support for changes in food handling practices from management and colleagues in the food industry; motivation to change behavior, belief system towards the occupational safety and health practice at the workplace, and the development of personal skills to apply the knowledge gained from a course (Seaman, 2010).

2.4.4 Social Cognitive Theory

Social cognitive theory (SCT) was known as Social Learning Theory (SLT), developed by infamous psychology professor Albert Bandura in 1960s. SCT provides an understanding of how human beings are dynamically influenced and influenced by our surroundings. It also explains how human behavior is regulated through observational learning and modeling processes and the influence of self-efficacy on the production of behavior. The characteristics components of SCT include reciprocal determinism, which refers to the dynamic and reciprocal interaction of person, environment and behavior. The other elements of SCT are the behavioral capability of an individual to perform a preventive behavior, observational learning from others, reinforcement for continuing or discontinuing the behavior, outcome expectation, and self-efficacy, as being depicted below (Glanz et al., 2008).

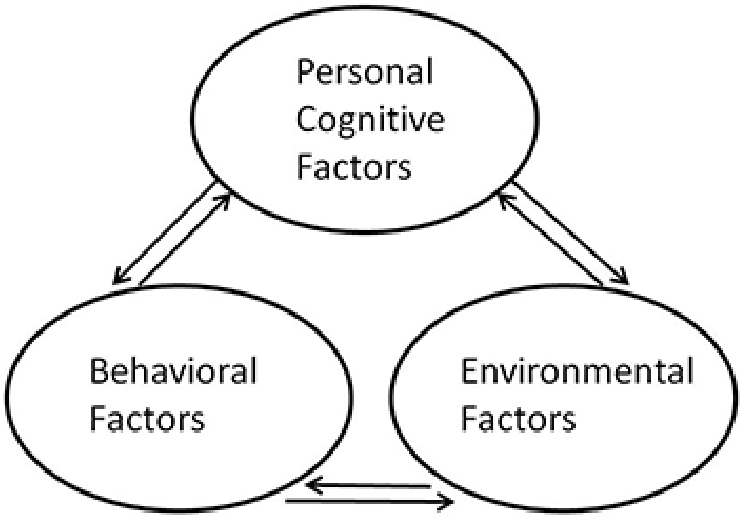

Figure 4. Social Cognitive Theory

Source: Luszczynska, A., & Schwarzer, R. (2015)

2.4.5 Knowledge, Attitude, and Practice (KAP) Theory

The KAP theory is another well-known health behavior change theory that explains the nature of behavior change among individuals. KAP theory suggests that an individual's behavioral changes can be divided into three processes: the acquisition of knowledge, the generation of attitudes, and the formation of behavior. Under this theory, knowledge can be defined as an individual understanding of any topic. The understanding will allow the individual to receive, retain and use specific information that at times mixed with individual experience and skills (Badran, 1995; Kaliyaperumal, 2004). Attitude, on the other hand, can be defined as participants' belief about the topic and propensity to react in a definitive action when facing a certain situation (Badran, 1995; Kaliyaperumal, 2004), and practice refers to how an individual demonstrates their knowledge and attitude (Badran, 1995; Kaliyaperumal, 2004).

The KAP theory presents the progressive relationship among knowledge, attitudes, and behavior: Knowledge and attitude are the driving forces for the change in practice. Only when people understand health-related knowledge and establish a positive attitude is it possible to form correct practice.

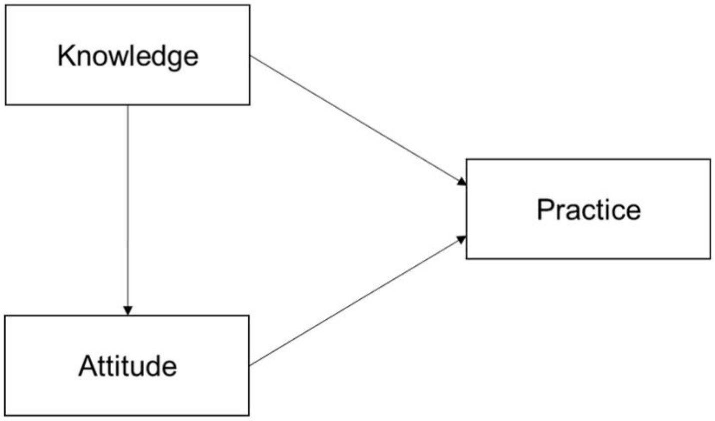

Figure 4. KAP Theory
Source: Zeng et. al. (2019)

In this study, the SCT was observed to be appropriate in the development of a theory-based intervention module as it provides a strong construct on the personal factors, environmental factors and behavioral factors on occupational safety and health among food handlers. It is aligned with the HBM that identified knowledge as an important modifying factor that influences individuals' beliefs and behaviors. It also suits the KAP theory that suggests that the initial process towards behavioral change or change in practice is an acquisition of knowledge. On the other hand, TRA explained the role of attitude that been characterized by an individual's belief about the outcome (practice) of certain behavior. It is again aligned with KAP theory, that propose knowledge as the foundation of behavior change, and belief and attitudes are the driving force of behavior change. Thus, in this study, the SCT component has been used as the theoretical framework of the module development. The HBM and KAP theory will then be adopted in exploring the KAP on occupational safety and health at food premises among food handlers. The conceptual framework of the presented theory is shown in Figure 5.

Figure 5. Conceptual Framework of TRIM-OSH based on SCT to improve KAP on OSH among food handlers working at food premises in Selangor

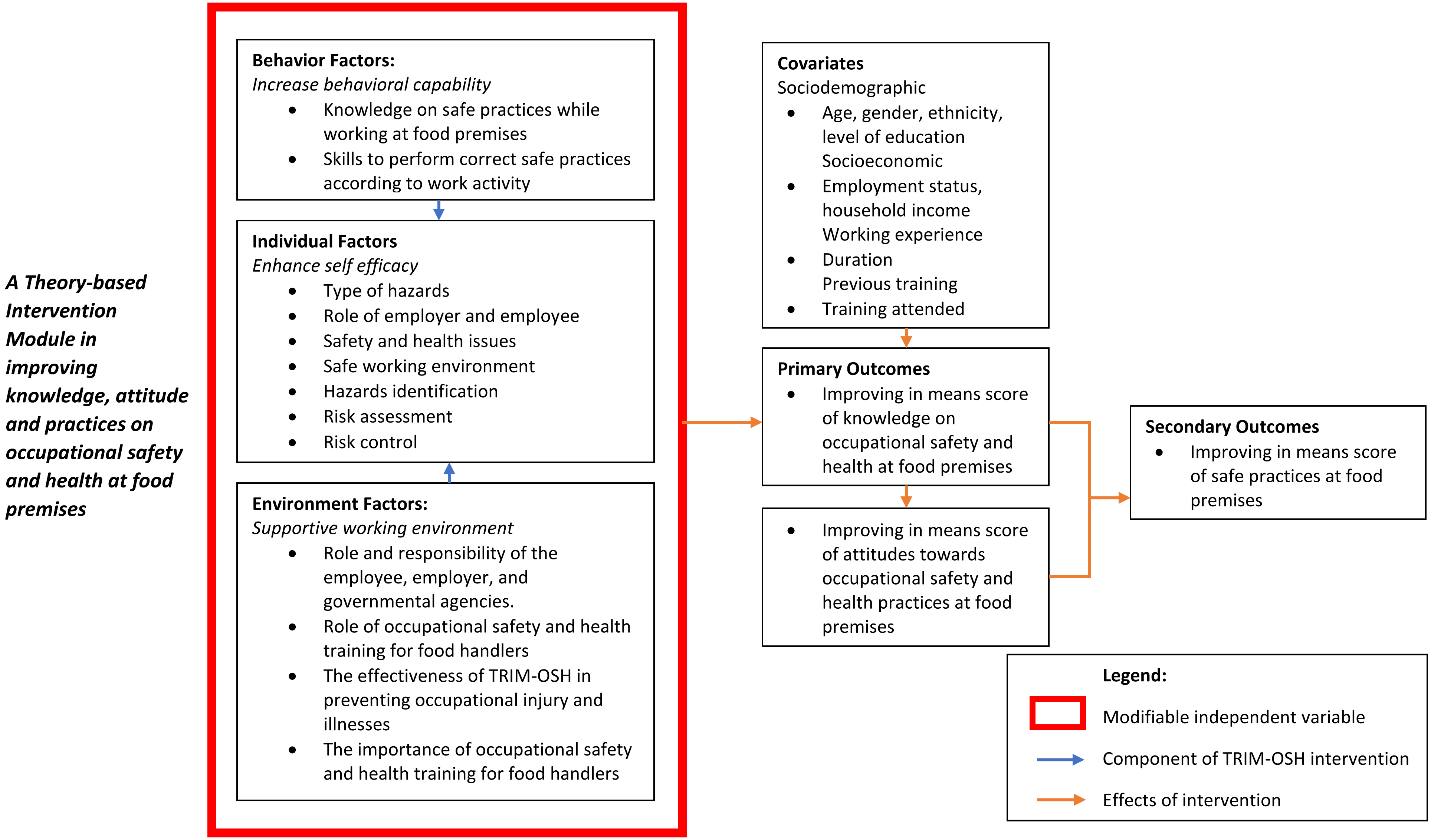

CHAPTER 3

METHODOLOGY

3.1 Study location

Due to MCO order imposed by the government on the verge of Covid-19 pandemic situation, 15% of the workers from the Services Sector reported that they had lost their job. The highest percentage of workers who lost their job were reported to come from the Food and Beverages industry at 35.4%, with 85.9% who remain in the industry reported having their income decreased with 61.6% reported having a reduction of more than 50%. In general, based on the DOSM special survey, 69.7% of workers who have been in the workforce for less than one year and 63.2% of those who have been in the workforce for a duration of 1-3 years were severely affected as they only have a financial saving that only, is able to survive for less than a month. This group of workers consists mainly of youth who just finish their education and enter the workforce from secondary or tertiary education at the age of 15-30-year-old.

In Malaysia, Selangor state has stood as the biggest contributor to Malaysia's GDP for years. Selangor is located on the right and west sides of Peninsular Malaysia, bounded by Perak in the north, Pahang in the east, Negeri Sembilan in the south, and Straits of Malacca in the West. Selangor has a total land area of 7,957km^2. The total population in Selangor is currently estimated at 6.53 million, with 3.40 million males and 3.13 million females. About 70% of the population are adults of age 20 and above. The average annual population growth rate in Selangor has decreased from 2016 to 2019, from 1.8% to 0.8%. Malay is the primary ethnicity with 3.60 million, followed by Chinese 1.57 million, Indian at 0.73 million, others 0.05 million, and non-Malaysian citizens 0.58 million. Selangor reported that it has achieved 91.4% urbanization in 2010 and is currently the most populous state in Malaysia (DOSM, 2021).

Regarding Covid-19 pandemic situation, Selangor recorded an increase in the unemployment rate among youth at 2.8% from 2019 to 2020 (6.1% to 8.9%), with the number of those who were unemployed in 2020 recorded at 101,800. This does not include adults outside the scope of youth from the age 15-30 but are still within the working range age up to 65-year-old. Considering these pieces of information, and the timing of the study duration, which will occur during the recovery period from post-Covid-19 pandemic where the food and beverages industry expected to catch up with the loss due to MCO, Selangor has been selected as the study location. The newly recruited labor force into the food and beverages industry in Selangor who undergo the compulsory Food Handler Training program as required by the ministry will also have the opportunity to involve in the formal training program on occupational safety and health provided during this research.

3.2 Study design

The study is a randomized controlled trial study. A single blinded, cluster randomized controlled trial study design will be conducted with pre-assessment and post-assessment on the intervention module. The respondents will be divided into two groups, intervention or control. The control group will receive a standard treatment and brochures related to occupational safety and health, while the intervention group will receive a theory-based intervention module on occupational safety and health among food handlers. The duration of the study is within 3-6 months.

3.2 Study population

The study population is food handlers of the working-age between 19-65 years old, B40 category, in Selangor, Malaysia.

3.3 Sampling population

3.3.1 Sampling unit

Sampling unit in this study is a registered food handler in Selangor under municipal in the working-age between 18-65 years old involved in the research and individually answers the questionnaire.

3.3.2 Inclusion criteria

The inclusion criteria are:

I. Malaysian citizen.
II. Working in Selangor, registered with municipal council
III. Involve in food and beverages industry.
IV. Age 18-65-year-old.
V. Consented to participate in the study

3.3.3 Exclusion criteria

The exclusion criteria are:

I. Unable to read and write in Malay language and English

3.4 Sampling frame

All food handlers of the legal working age between 19-65 years old, B40 category, in Selangor, Malaysia.

3.5 Sampling technique

There are 324 Food Handler Training Schools in Selangor located in nine districts. This study will use a simple random sampling technique to select the sampling location. Four districts will be randomly selected. Once the district is determined, the selection of the Food Handler Training School within the district will proceed by using a similar method and subsequently assigned. The flow of the sampling technique is shown in the figure below:

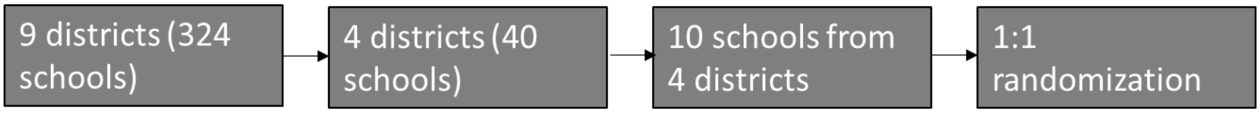

Figure 5. Cluster random sampling flow chart.

Cluster randomization will be used to ensure administrative efficacy, lessened risk of experimental contamination, and enhancement of subject compliance. The participants attending the food handler training program will be invited into the study via invitation and assessed for eligibility. The unit of randomization will be on a 1:1 basis in either the control or intervention group.

An independent research staff, blinded as to whether they were nominating within the control or an intervention group and unaware which of their participants had consented to participate.

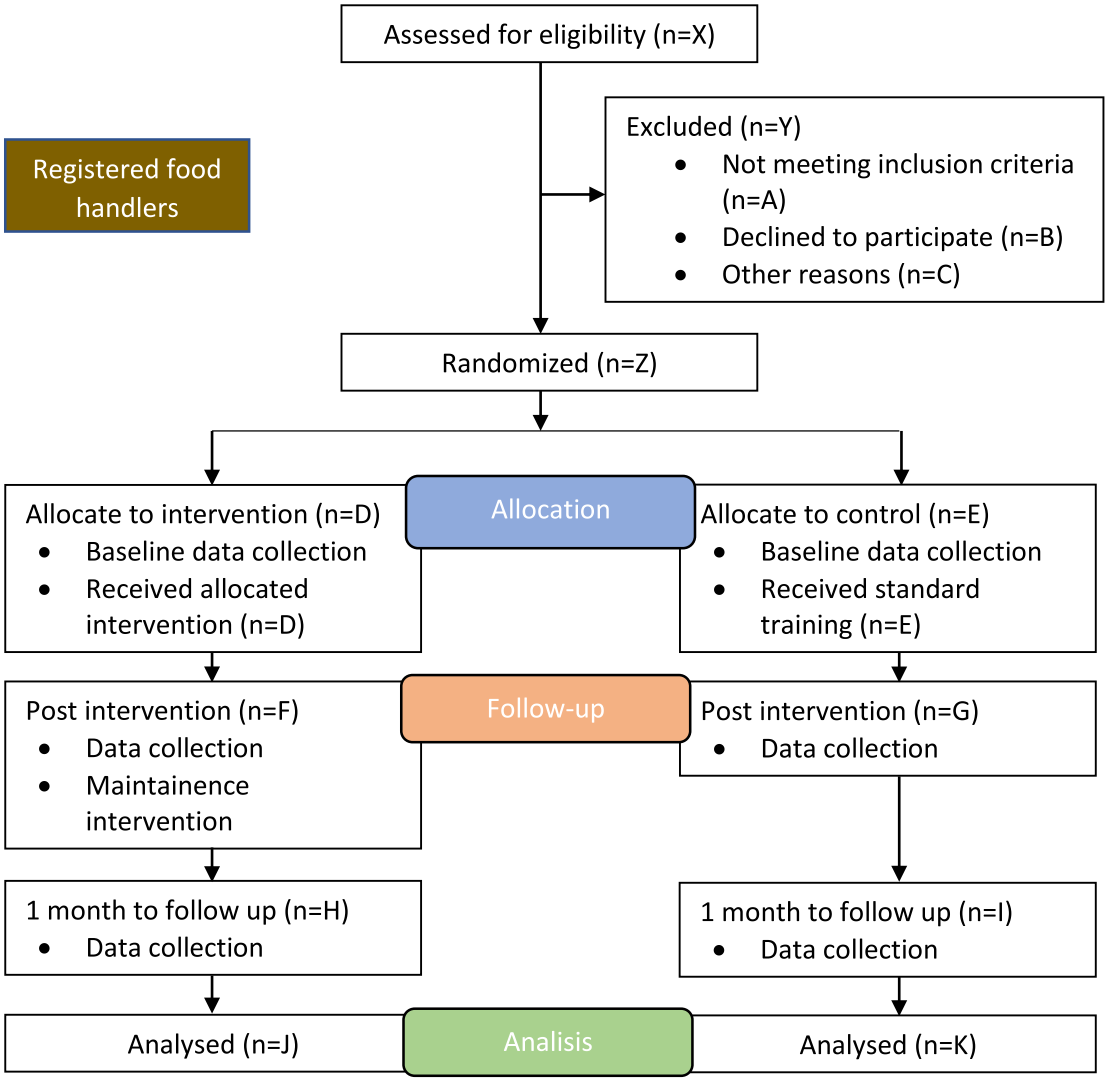

Figure 6. Proposed research Flow Chart

3.6 Sample size

The sample size calculation in this study was based on the targeted primary outcomes, such as knowledge, attitude, and practice of safety and health. A previous power analysis specified an enrolment target of 20 clusters of six participants per condition. Provided that the average attrition rate in previous intervention studies was 20%, conservative estimation will be conducted on the attrition at 20% for each testing time point when the target sample size is designed. Given that the total time points in this study requirement are three times follow up after the baseline data collection, the required sample size comprises 20 clusters of 12 participants per group (N = 240). To encourage retention, lottery-style drawings will be carried out for participants who complete each data

collection phase. Retention bonus pay will be offered for those who complete all four data collection phases.

3.6 Variables

3.6.1 Dependent Variable

The primary outcome of this study is:

i. The changes in the means score of knowledge on occupational safety and health at food premises.
ii. The changes in the means score of attitudes towards occupational safety and health practices at food premises

The secondary outcome of this study is:

iii. The changes in means score of safe practices at food premises from baseline to endpoint and,
iv. The maintenance of knowledge, good attitude, and safe practices at work from baseline to endpoint.

3.6.2 Independent Variables

The independent variables in this study:

A theory-based intervention module that being developed and implemented to examine on its effects on improving KAP score and maintaining good KAP score among food handlers attending the programme.

Covariates:

I. Sociodemographic factors (age, gender, ethnicity and level of education)
II. Working experience (number of years in labor force, and previous employment history)
III. Training attendance (participation in occupational safety and health training)

3.7 Instrument

A self-administrated questionnaire in 2 language versions (English and Malay) in Google Form format and hardcopy format will be used in this study. The questionnaire used in the study is a new instrument that will be developed based on the latest global guidelines, the instructions set by Iran's Ministry of Health and Education (MOH), and literature reviews, as well as the WHO, CDC, and other guidelines in the field of occupational safety and health-related to the food industry with the component of KAP. The questionnaire will be divided into four parts as below:

Part 1: Personal information.
Consist of questions of sociodemographic data (age, gender, ethnicity and level of education), employment status, income, working experience (number of years in labor force, previous and previous employment history), and training attendance (participation in occupational safety and health training).

Part 2: Knowledge on occupational safety and health among food handlers
Consist of questions on knowledge on occupational safety and health among food handlers.

Part 3: Attitude towards occupational safety and health among food handlers
Consist of the questions on the attitude on occupational safety and health among food handlers

Part 4: Practice of occupational safety among food handlers

Consist of questions about the practice on occupational safety and health among food handlers

3.8 Development of theory-based intervention module

The theory-based intervention on occupational safety and health among food handlers will be developed in Malay and English under the supervision of the supervisory committee. The intervention module will be developed for food handlers using an educational intervention model adopted from SCT. The validation of the module training will be based on the review and recommendation of the panel of content experts in occupational safety and health from the Faculty of Medicine and Health Sciences, UPM, to ensure that the content is appropriate and suitable for the intervention module.

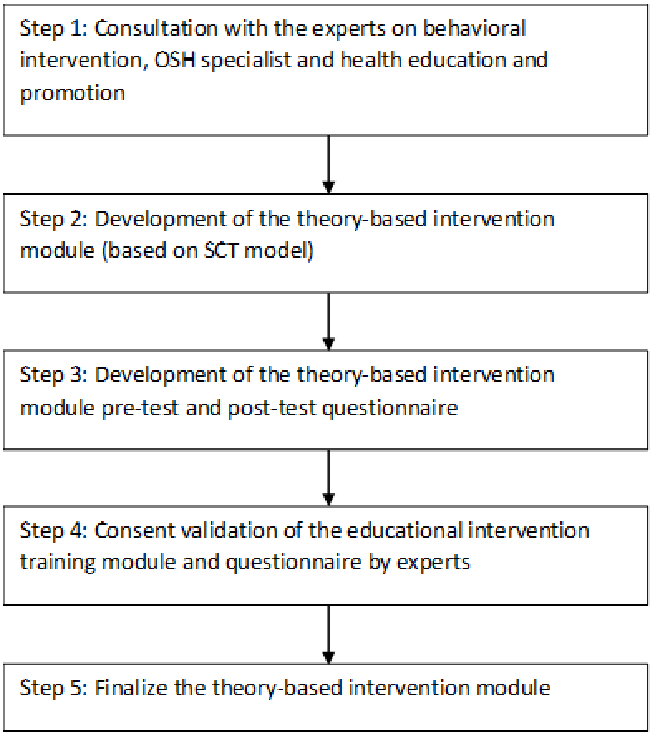

Figure 7. Schematic diagram of the development of a theory-based intervention module to improve means KAP score and maintenance of KAP among food handlers.

3.9 Data collection methods

The data collection will occur in the selected districts in Selangor, Malaysia, within a given time frame. The questionnaire will be distributed to selected respondents in the selected Food Handler Training School. All participants who agree to participate in the study will be consented by agreeing to the agreement statement prior study begins. The questionnaire will be given in the Google Form format for the participants to access the form with the response collected immediately upon completion before and immediately after training. The participants will be contacted after one-month post-training for the reassessment of the KAP score. If the accessibility of the Google Form is not available, the same questionnaire in the hardcopy format will be given and collected on the same day at all mentioned events.

3.10 Data analysis

The collected data and will be analyzed using IBM Statistical Package for Social Science (SPSS) version 25 involving descriptive and inferential statistics. Descriptive statistics will be used to describe the characteristics of participants. Whilst, the descriptive analysis for continuous variables with mean, mode, median and percentage will be analyzed.

The bivariate analysis of Chi-square will be used to measure associations between two categorical variables. A generalized Linear Mixed Model (GLMM) then will be conducted to determine the effects of sociodemographic factors (age, gender, ethnicity, level of education), socioeconomic (employment status, household income), working experience (duration) and previous training (training attended) on KAP on occupational safety and health at food premises among food handlers pre and post intervention within and between study groups.

In further analysis, the effects of SCT constructs component as the base of the theory-based intervention module which is behavior, individual and environment factors on KAP on occupational safety and health at food premises among food handlers pre and post intervention within and between study groups will be determined.

The effects that are going to be determined is fixed and random effects. The fixed effect is factors that the only level under consideration are continued in coding those effects. For example, both male and female genders are included in the factor sex; other examples as different ethnicities are included in the factor ethnicity group. On the other hand, the random effect is the factors where its effects are random, where they are assumed to be drawn randomly from a distribution during the data-generating process. In this study, the study sample is a random sample of the target population of food handlers at food premises and the study needs to measure KAP score of the food handlers a few times.

GLMM can incorporate both fixed and random effects. Individual's response at baseline (intercept) is allowed to differ across subjects by releasing the random effect of intercept. The change in response over time is also allowed to differ across subjects by releasing the random effect of a linear slope. By adding the random effects in the model, GLMM provides estimated parameters (fixed effect) and individual variability of these parameters around the population trend (Ellison, 2017).

All results of log-transformed data considered underlying data transformation in result interpretation. The confidence interval will be at set at 95% for mean estimations. The level of significance, alpha (α), will be set at 0.05. A p-value of less than 0.05 pertained to the decision rule. The dependent variables measured in this study are the outcome (primary and secondary) and effectiveness of the SCT constructs as the base of theory-based intervention module in occupational safety and health training among food handlers.

3.11 Quality control

The instrument will test for validity and reliability as follows:

I. Face validity: A pre-test will be conducted among ten food handlers' staffs from Hospital Perubatan Universiti Putra Malaysia. The questionnaire will be revised based on their feedback and comment.
II. Content validity: The questionnaire will be discussed among two specialists in Public Health Medicine for assurance.
III. Reliability: A test-retest of the questionnaire will be conducted among adult at the age of 19-65 at the selected Food Handler Training in Selangor, Malaysia. The data will be assessed using kappa statistics (K).

3.12 Ethical approval

Ethical approval will be obtained from the Ethics Committee for Research involving Human Subjects of University Putra Malaysia (JKEUPM). A consent form from each participant will be obtained upon agreement to participate in the study before answering the questionnaire.

3.13 Operational definition of variables

Table 5: Independent variable and dependent variable

Independent Variable	Dependent Variable
**Sociodemographic**	
Age	KAP score on occupational safety and health among food handlers
Gender	
Ethnicity	
Level of education	

Working experience	
Training Attendence	
**KAP**	
Knowledge	KAP score on occupational safety and health among food handlers
Attitude	
Practice	

In the second section, there will be multiple-choice items focused on the knowledge, attitude, and self-reported practice of the food handlers working at food premises (Cunha et al., 2019; Kunadu et al., 2016; Majowicz et al., 2015). The knowledge questions will be comprised of items to be scored as zero scores for “false” and “I do not know” answers and one score for “true” answers, which classified the scores as low (<2), moderate (Angulo & Jones, 2006; World Health Organization, 2020), and good (Jain, 2020).

The attitude consisted of three items to be rated on five-point Likert scales: “completely disagree,” “disagree,” “unsure” “agree,” and “strongly agree,” each weighing 1–5 scores, respectively. The total scores were classified as strongly negative (<5), negative (5–10), positive (11–12), and strongly positive (13–15). Some questions were reversed to diminish the possible bias of giving a single similar response in all the items.

As for practice, there will be suggested items rated as five-point Likert-item questions with the responses of never, rarely, sometimes, often, and always, each weighing 1–5 scores, respectively. Their self-reported practice scores of them were classified as weak (<40), acceptable (40–50), and desirable (51–60). In addition, the observed practices presented a slight correlation with the self-reported practices in a previous study (Cunha et al., 2019).

The last section included three questions about the participants’ source of information, their need for education, and how they can increase their knowledge

of this issue. Also, they were asked to comment on (a) What they most liked about the course, (b) How it could be improved, and (c) What they will do differently at work as a result of their training.

CHAPTER 4

RESULTS

4.1 Results Dummy Table

Table A. Baseline comparison of sociodemographic, socioeconomic, working experience, and training attendance characteristics between-group (n=).

	Group median (IQR) / n (%)		*Statistical Test*	*p-value*
	**Control**	**Intervention**		
**Age (years)**				
**Gender**				
Male				
Female				
**Ethnicity**				
Malay				
Chinese				
Indian				
Others				
**Educational status**				
Primary school				
Secondary school				
SPM/STPM/Matriculation/Diploma				
First degree				
Second degree and third degree				
No formal education				
**Employment status**				
Government servant				
Private company				
Self-employed				
Student				
Unemployed				
**Monthly individual income (RM)**				
**Monthly household income (RM)**				
**Previous working experience**				
Yes				
No				
**Length of experience (years)**				
**Previous training attendence**				
Yes				
No				
**Typhoid injection status**				
Yes				
No				

Table B. Between-group difference of total score of self-efficacies, behavioral capacity, and supportive working environment on occupational safety and health at food premises.

	Group median (IQR)		
	**Baseline**	**Post-intervention**	**1-month**
**The total score of self efficacies**			
Control			
Intervention			
Man-Whitney U Test			
p-value			
**The total score of behavioral capacity**			
Control			
Intervention			
Man-Whitney U Test			
p-value			
**The total score of supportive working environment**			
Control			
Intervention			
Man-Whitney U Test			
p-value			

Significant at p<0.05

Table C. Within intervention group difference of total score of self-efficacies on occupational safety and health at food premises.

**Self-efficacy on occupational safety and health at food premises**	**Mean ± SD**	**F***	**p-value****
Within Intervention group.			
Baseline			
Post-intervention			
1-month			
Within Control Group			
Baseline			
Post-intervention			
1-month			

*Repeated measure ANOVA
**Bonferroni adjustment
***Significant at p<0.05

Table D. Within intervention group difference of total score of behavioral capacity on occupational safety and health at food premises.

**Behavioral capacity on occupational safety and health at food premises**	**Mean ± SD**	**F***	**p-value****
Within Intervention group.			
Baseline			

Post-intervention			
1-month			
Within Control Group			
Baseline			
Post-intervention			
1-month			

*Repeated measure ANOVA
**Bonferroni adjustment
***Significant at p<0.05

Table E. Within intervention group difference of total score of supportive working environement on occupational safety and health at food premises.

**Supportive environment on occupational safety and health at food premises**	**Mean ± SD**	**F***	**p-value****
Within Intervention group.			
Baseline			
Post-intervention			
1-month			
Within Control Group			
Baseline			
Post-intervention			
1-month			

*Repeated measure ANOVA
**Bonferroni adjustment
***Significant at p<0.05

Table F. Fixed Coefficent of variables for self efficacy on occupational safety and health at food premises

**Variable**	**Coefficients**	**Std. Error**	***t***	**p-value**	**95% CI**	
					**Lower**	**Upper**
**Group**						
Intervention						
Control						
**Time**						
Baseline						
Post-intervention						
1-month						
**Education**						
Primary						
Secondary						
Tertiary						

ACKNOWLEDGEMENTS

We would like to thank the Director General of Health Malaysia for his permission to publish this article. Special thanks to Professor Dr. Zamberi Sekawi, the Dean of Faculty of Medicine and Health Sciences, Universiti Putra Malaysia, for allowing us to publish this paper. We also would like to thank the Director General of Health Malaysia for his permission to publish this article and all the related authorities for the study approval.

REFERENCES

Alemayehu, T., Aderaw, Z., Giza, M., & Diress, G. (2021). Food safety knowledge, handling practices and associated factors among food handlers working in food establishments in debre markos town, northwest ethiopia, 2020: Institution-based cross-sectional study. *Risk Management and Healthcare Policy*, *14*, 1155–1163. https://doi.org/10.2147/RMHP.S295974

Badran, I. G. (1995). Knowledge, attitude and practice the three pillars of excellence and wisdom: a place in the medical profession.

Bandura, A. (1997). Self-efficacy: The exercise of control. New York: Freeman.

Bintsis, T. (2017). Foodborne pathogens. *AIMS Microbiology*, *3*(3), 529–563. https://doi.org/10.3934/microbiol.2017.3.529

Bovenzi, M., Schust, M., & Mauro, M. (2017). An overview of low back pain and occupational exposures to whole-body vibration and mechanical shocks. *Medicina Del Lavoro*, *108*(6), 419–433. https://doi.org/10.23749/mdl.v108i6.6639

Campbell, M., Fitzpatrick, R., Haines, A., Kinmonth, A. L., Sandercock, P., Spiegelhalter, D., & Tyrer, P. (2000). Framework for design and evaluation of complex interventions to improve health. BMJ, 321, 694–696. doi:10.1136/bmj.321.7262.694

Clayton, D. A., & Griffith, C. J. (2008). Efficacy of an extended theory of planned behaviour model for predicting caterers' hand hygiene practices. *International Journal of Environmental Health Research*, *18*(2), 83–98. https://doi.org/10.1080/09603120701358424

Davis, R., Campbell, R., Hildon, Z., Hobbs, L., & Michie, S. (2015). Theories of behaviour and behaviour change across the social and behavioural sciences: a scoping review. Health psychology review, 9(3), 323-344.

De Matteis, S., Heederik, D., Burdorf, A., Colosio, C., Cullinan, P., Henneberger, P. K., Olsson, A., Raynal, A., Rooijackers, J., Santonen, T., Sastre, J., Schlünssen, V., Tongeren, M. Van, & Sigsgaard, T. (2017). Current and new challenges in occupational lung diseases. *European Respiratory Review*, *26*(146), 1–15. https://doi.org/10.1183/16000617.0080-2017

DOSH. (2021). *Occupational Poisoning and Diseases Statistics 2019*. *March*, 12000. https://www.dosh.gov.my/index.php/ms/statistik/occupational-diseases-statistic/3869-2019/file

DOSM. (2019). Department of statistics Malaysia: Press release annual economic statistics 2018 Food and Beverage services. *Department of Statistics Malaysia*,

March, 1–2.

DOSM. (2021). *Selangor @ a Glance*. Department of Statistic Malaysia. https://www.dosm.gov.my/v1/index.php?r=column/cone&menu_id=eGUyTm9RcEVZSllmYW45dmpnZHh4dz09

Dudeja, P., & Singh, A. (2017). Chapter 21 – Food handlers. In *Food Safety in the 21st Century*. Elsevier Inc. https://doi.org/10.1016/B978-0-12-801773-9/00021-2

Ellison, M. C. (2017). Repeated Measures Design with Generalized Linear Mixed Models for Randomized Controlled Trials, by Toshiro Tango. Journal of Biopharmaceutical Statistics, 27(6), 1121–1122. https://doi.org/10.1080/10543406.2017.1362625

Flanders Investment & Trade Malaysia Office. (2020). Food & Beverage Industry Report Malaysia 2020. *Tapio Management Advisory Sdn Bhd,* 27.

Frizelle, G. M. (1995). Health education models and food hygiene education. *Journal of the Royal Society of Health*, *1*(2), 165–168.

Glanz, K., Rimer, B. K., & Viswanath, K. (Eds.). (2015). Health behavior: Theory, research, and practice. John Wiley & Sons.

Goh, Y. M., & Chua, S. (2016). Knowledge, attitude and practices for design for safety: A study on civil & structural engineers. *Accident Analysis and Prevention*, *93*, 260–266. https://doi.org/10.1016/j.aap.2015.09.023

Has, S. M. C., Jaafar, S. N. A., & Chilek, T. Z. T. (2018). An assessment on pre-and post-food hygiene training on food safety's Kap level among food handlers in Kuala Terengganu and Kuala Nerus. *Malaysian Applied Biology*, *47*(4), 61–69.

Isara, A. R., & Isah, E. C. (2009). Knowledge and practice of food hygiene and safety among food handlers in fast food restaurants in Benin City, Edo State. The Nigerian postgraduate medical journal, 16(3), 207–212.

Kaliyaperumal, K. I. E. C. (2004). Guideline for conducting a knowledge, attitude and practice (KAP) study. AECS illumination, 4(1), 7-9.

Laws of Malaysia Act 281 Food Act. (2006). *Laws of Malaysia Act 281 Food Act 1983*. 1–24.

Luszczynska, A., & Schwarzer, R. (2015). Social cognitive theory. Fac Health Sci Publ, 225-51.

U.S. DOL, BLS(a)(2020). "Table 2 - Number of cases - detailed industry level. Summary tables. 2022. Washington, DC: U.S. DOL, BLS, 2020. Apr. 3, 2022. https://www.bls.gov/iif/oshwc/osh/os/summ2_00_2020.xlsx

U.S. DOL, BLS(b)(2020). “Table R1. Number of nonfatal occupational injuries and illnesses involving days away from work by industry and selected natures of injury or illness, private industry, 2020”. Case circumstances and worker characteristics for injuries and illnesses involving days away from work by Industry. 2022. Washington, DC: U.S. DOL, BLS, 2020. Apr. 3, 2022. https://www.bls.gov/iif/oshwc/osh/case/cd_r1_2020.xlsx

U.S. DOL, BLS(c)(2020). "Table R9. Number of nonfatal occupational injuries and illnesses involving days away from work by occupation and selected natures of injury or illness, private industry, 2020". Case circumstances and worker characteristics for injuries and illnesses involving days away from work By Occupation. 2022. Washington, DC: U.S. DOL, BLS, 2020. Apr. 3, 2022. https://www.bls.gov/iif/oshwc/osh/case/cd_r9_2020.xlsx

U.S. DOL, BLS(d)(2020). "Table R4. Number of nonfatal occupational injuries and illnesses involving days away from work by industry and selected events or exposures leading to injury or illness, private industry, 2020". Case circumstances and worker characteristics for injuries and illnesses involving days away from work by Industry. 2022. Washington, DC: U.S. DOL, BLS, 2020. Apr. 3, 2022. https://www.bls.gov/iif/oshwc/osh/case/cd_r4_2020.xlsx

Lwanga, S. K., & Lemeshow, S. (1991). Sample size determination in health studies: a practical manual. *World Health Organization.*

Makanan, 1983 Akta. (2009). *Bab 1 – Kewajipan tuanpunya, pemunya atau penghuni premis makanan 8.* 1–60.

Malaysian Investment Development Authority. (2019). Ideal Prospects, Immense Opportunities Diverse Resources. *Malaysian Investment Development Authority (MIDA)*, 13. http://www.mida.gov.my/home/administrator/system_files/modules/photo/uploads/20180903103354_Food Industry 2018_V4.pdf

Mirza, R., Kirchner, D. B., Dobie, R. A., & Crawford, J. (2018). Occupational Noise-Induced Hearing Loss. *Journal of Occupational and Environmental Medicine*, *60*(9), e498–e501. https://doi.org/10.1097/JOM.0000000000001423

MOH. (2014). Kursus Latihan Pengendali Makanan. *Ministry of Health Malaysia*, 1–35. http://fsq.moh.gov.my/v6/xs/page.php?id=222

Mohd Ishanuddin, N., Sukadarin, E. H., Abdul Aziz, H., & Zakaria, J. (2019). An Investigation of Knowledge, Attitude and Practice of Occupational Safety and Health (OSH) on Safety Climate at Workplace in Manufacturing Industry. *Journal of*

Occupational Safety and Health, *16*(2), 21–29.

Mt, A., Bmt, S., & Mn, D. (2021). *Impact of an Intervention Program on Improvement of Knowledge, Attitudes, Practices (KAP) on Food and Occupational Health Safety Among Cattle Abattoir Workers in Malaysia Objective: Food and occupational safety and proper meat handling are some of th*. *7*(1), 19–24.

Nee, S. O., & Sani, N. A. (2011). Assessment of Knowledge, Attitudes and Practices (KAP) Among food handlers at residential colleges and canteen regarding food safety. *Sains Malaysiana*, *40*(4), 403–410.

Petricciani, J. (2009). Biologicals. *Biologicals*, *37*(4), 270. https://doi.org/10.1016/j.biologicals.2009.02.001

Seaman, P. (2010). Food hygiene training: Introducing the Food Hygiene Training Model. *Food Control*, *21*(4), 381–387. https://doi.org/https://doi.org/10.1016/j.foodcont.2009.08.005

Thetkathuek, A., Yingratanasuk, T., Jaidee, W., & Ekburanawat, W. (2015). Cold exposure and health effects among frozen food processing workers in Eastern Thailand. *Safety and Health at Work*, *6*(1), 56–61. https://doi.org/10.1016/j.shaw.2014.10.004

Tomoda, S., & International Labour Office. Industrial Activities Branch. (1993). *Occupational safety and health in the food and drink industries*.

Unit Perancang Ekonomi, J. P. M. (2019). *Table 1 . 4 : Employed persons by industry , Malaysia , 1982 – 2000 Tahun Year Jumlah Total Industri Industry*. *2000*, 1982–2019.

Zulkarnain, M., Flora, R., Faisya, A. F., Martini, S., & Aguscik. (2020). *Dehydration Index and Fatigue Level of Workers Laboring in Heat-Exposed Environments*. *25*(Sicph 2019), 164–168. https://doi.org/10.2991/ahsr.k.200612.022

Zeng, Y., Hu, X., Li, Y., Zhen, X., Gu, Y., Sun, X., & Dong, H. (2019). The quality of caregivers for the elderly in long-term care institutions in Zhejiang Province, China. MDPI. Retrieved April 20, 2022, from https://doi.org/10.3390/ijerph16122164

APPENDICES

THEORY-BASED INTERVENTION MODULE ON OCCUPATIONAL SAFETY AND HEALTH AT FOOD PREMISE (TRIM-OSH) OUTLINE

IDENTIFIED PANEL OF EXPERTS

(A) UNIVERSITI PUTRA MALAYSIA

1. **Prof. Dr. Kulanthayan K.C. Mani**
   Pensyarah Perubatan (Kesihatan Awam)
   Jabatan Kesihatan Komuniti
   Fakulti Perubatan dan Sains Kesihatan

2. **Prof. Madya Dr. Ahmad Azuhairi Ariffin**
   Pensyarah Perubatan (Kesihatan dan Keselamatan Pekerjaan)
   Jabatan Kesihatan Komuniti
   Fakulti Perubatan dan Sains Kesihatan

3. **Dr. Suhainizam Muhamad Saliluddin**
   Pensyarah Perubatan (Kesihatan dan Keselamatan Pekerjaan, Kesihatan Persekitaran)
   Jabatan Kesihatan Komuniti
   Fakulti Perubatan dan Sains Kesihatan

4. **Prof. Madya Dr. Anita Abd Rahman**
   Pensyarah Perubatan (Kesihatan dan Keselamatan Pekerjaan, Kesihatan Awam)
   Jabatan Kesihatan Komuniti
   Fakulti Perubatan dan Sains Kesihatan

5. **Dr Zawiah Mansor**
   Pensyarah Perubatan (Kesihatan Awam)
   Jabatan Kesihatan Komuniti
   Fakulti Perubatan dan Sains Kesihatan

(B) UNIVERSITI KEBANGSAAN MALAYSIA

1. **Prof. Madya Dr Mohd Rohaizat bin Hassan**
   Pensyarah Perubatan (Kesihatan Awam)
   Jabatan Kesihatan Masyarakat
   Fakulti Perubatan

2. **Professor Dr Zaleha binti Md Isa**
   Pensyarah Perubatan (Keselamatan dan Kesihatan Makanan)
   Jabatan Kesihatan Masyarakat

Fakulti Perubatan

(C) UNIVERSITI TEKNOLOGI MARA

1. Prof. Madya Dr Nazri bin Che Dom
Pensyarah Perubatan (Kesihatan Awam)
Jabatan Kesihatan Persekitaran
Fakulti Perubatan

INTERVENTION MODULE OUTLINE

FIRST SESSION (1 HOUR)

MAIN OBJECTIVE:

1. To enhance self efficacy among food handlers on occupational safety and health at food premise

SPECIFIC OBJECTIVE:

1. To introduce food handlers on common type of hazards at food promises
2. To introduce food handlers on common occupational safety and health issues among food handlers
3. To educate food handlers on safe working environment at food premises
4. To emphasize on role of employer and employee in regards of occupational safety and healh at food premise
5. To educate food handlers on appropriate risk assessments and risk controls towards identified hazards at food premises

CONTENT OUTLINE:

1. Type of hazards
2. Role of employer and employee
3. Safety and health issues
4. Safe working environment
5. Hazards identification
6. Risk assessment
7. Risk control

MODE OF DELIVERY:

1. Web-based online course
2. Power point presentation
3. Video demonstration
4. Quiz

SECOND SESSION (1 HOUR)

MAIN OBJECTIVE:

1. To potrait supportive working environment on occupational safety and health at food premises among food handlers

SPECIFIC OBJECTIVE:

1. To instill the importance of shared responsibility between employer, employee, and governmental agency in ensuring supportive working environment towards occupational safety and health at food premises
2. To emphasize the role and importance of an effective training for food handlers in preventing occupational injury and illnesses

CONTENT OUTLINE:

1. Role and responsibility of the employee, employer, and governmental agencies.
2. Role of occupational safety and health training for food handlers
3. The effectiveness of TRIM-OSH in preventing occupational injury and illnesses
4. The importance of occupational safety and health training for food handlers

MODE OF DELIVERY:

1. Web-based online course
2. Power point presentation
3. Video demonstration
4. Quiz

THIRD SESSION (1 HOUR)

MAIN OBJECTIVE:

1. To increase behavioral capabilities on occupational safety and health practices among food handlers at food premises

SPECIFIC OBJECTIVE:

1. To impart knowledge on safe practices while working at food premises based on given circumstances
2. To demonstrate on skills to perform correct safe practices based on given work activity

CONTENT OUTLINE:

1. Knowledge on safe practices while working at food premises
    a. Identification of wrong practices

2. Skills to perform correct safe practices according to work activity
    a. Lifting heavy object
    b. Handling of sharp objects
    c. Handling of machine

MODE OF DELIVERY:

1. Web-based online course
2. Power point presentation
3. Video demonstration
4. Quiz
